# Theory of relaxor-ferroelectricity

**DOI:** 10.1038/s41598-020-61911-5

**Published:** 2020-03-19

**Authors:** Li-Li Zhang, Yi-Neng Huang

**Affiliations:** 1 National Laboratory of Solid State Microstructures, School of Physics, Nanjing University, Nanjing, China; 2 Xinjiang Laboratory of Phase Transitions and Microstructures in Condensed Matters, College of Physical Science and Technology, Yili Normal University, Yili, China

**Keywords:** Mathematics and computing, Physics

## Abstract

Relaxor-ferroelectrics are fascinating and useful materials, but the mechanism of relaxor-ferroelectricity has been puzzling the scientific community for more than 65 years. Here, a theory of relaxor-ferroelectricity is presented based on 3-dimensional-extended-random-site-Ising-model along with Glauber-dynamics of pseudospins. We propose a new mean-field of pseudospin-strings to solve this kinetic model. The theoretical results show that, with decreasing pseudospin concentration, there are evolutions from normal-ferroelectrics to relaxor-ferroelectrics to paraelectrics, especially indicating by the crossovers from, (a) the sharp to diffuse change at the phase-transition temperature to disappearance in the whole temperature range of order-parameter, and (b) the power-law to Vogel-Fulcher-law to Arrhenius-relation of the average relaxation time. Particularly, the calculated local-order-parameter of the relaxor-ferroelectrics gives the polar-nano-regions appearing far above the diffuse-phase-transition and shows the quasi-fractal characteristic near and below the transition temperature. We also provide a new mechanism of Burns-transformation which stems from not only the polar-nano-regions but also the correlation-function between pseudospins, and put forward a definition of the canonical relaxor-ferroelectrics. The theory accounts for the main facts of relaxor-ferroelectricity, and in addition gives a good quantitative agreement with the experimental results of the order-parameter, specific-heat, high-frequency permittivity, and Burns-transformation of lead magnesium niobate, the canonical relaxor-ferroelectric.

## Introduction

65 years after the discovery of so-called relaxor-ferroelectrics (RFEs)^1^, this manuscript promises to deliver the still missing theory of relaxor-ferroelectricity [Supplementary Information (SI) 1]^2–10^. For the existing phase-transition theories of normal-ferroelectrics are based on both structure and component homogeneity^11–13^, theoretically, the main difficulty in describing relaxor-ferroelectricity originates from RFEs being component-disordered although structure-ordered, i.e. disordered components on crystal lattices^14–19^. In fact, understanding how the component disorder on lattices leads to novel properties is an outstanding scientific challenge for a broad class of materials that include not only RFEs, but also spin glasses^20^, superelastic strain glasses (shape-memory alloys)^21^, colossal magnetoresistance manganites, and some superconductors^22^.

The best-known member of the RFE family is the disordered perovskite crystal PbMg_1/3_Nb_2/3_O_3_ (PMN), for which 27 years ago a plausible interpretation of its diffuse-phase-transition (DPT) was proposed by Westphal *et al*.^3^. Fluctuations of random-internal-electric-field (RIEF) emerging from the quenched charge disorder of the RFE are stabilizing the typical disordered polar nanodomain state. This disordering mechanism convinced sceptical experts at the latest thanks to a favorable review of Cowley *et al*.^16^. The subsequent lattice-dynamical theory of Arce-Gamboa and Guzmán-Verrí^10^, involving a Gaussian distribution of RIEF yielded indeed the predicted nanodomain state with anisotropic and power-law correlations.

A similar change of mind occurred with regard to the quenched random-internal-stress-field (RISF) in isovalent relaxors such as BaZr_x_Ti_1−x_O_3_ (BZ_x_T_1−x_). Experimental evidence of quenched random local displacements of the ferro-active Ti^4+^ ions in the RFE state^23^ motivated Kleemann^24^ to propose RISF being due to randomly distributed large Zr^4+^ and Sn^4+^ ions, respectively, and thus to give rise to relaxor behavior also in such systems.

Starting from this level of knowledge, the present authors have developed a theory of relaxor-ferroelectricity within a 3-dimensional-random-site-Ising-model (3D-RSIM)^25–28^ involving Glauber dynamics^29,30^ of pseudospins (PSs. Equivalence of the orientational motion of permanent electric dipoles to spins)^11,12^, and the interaction of PSs with both RIEF and RISF^3,4,16,24^. Meanwhile, we propose a new mean-field of pseudospin-strings to solve this kinetic model.

The main facts of relaxor-ferroelectricity, i.e. the novel phase-transition phenomenon of RFEs, are: (i) As a function of temperature, (a) the frequency-dependent peak of permittivity, with a broad distribution of relaxation time and the average relaxation time varying as the Vogel-Fulcher-law^31–33^, (b) the diffuse change of spontaneous-polarization (order-parameter)^34–38^, and particularly, the quasi-fractal characteristic of the local-spontaneous-polarization (local-order-parameter) as well as its variation^17,18^, (c) the small broad peak of specific-heat^39–41^, and (d) Burns-transformation^42,43^ and the corresponding polar-nano-regions (PNRs) appearing far above the DPT^44–47^; and (ii) With varying components, the evolutions between normal-ferroelectrics ↔ RFEs ↔ paraelectrics^48–51^. Our theory can account for these facts, and in addition gives a good quantitative agreement with the experimental results of the order-parameter, specific-heat, high-frequency permittivity, and Burns-transformation of PMN, the generally viewed canonical RFE^52,53^, which is convincing evidence that the theory is essentially correct.

## Results

### Theory of relaxor-ferroelectricity

In view of that, (a) RFEs have component disorder on crystal lattices^14,17,19^, (b) BZ_x_T_1−x_^49,51^ and Sr_x_Ba_1−x_Nb_2_O_6_^48,50^ evolve from normal-ferroelectrics to RFEs with increasing x, (c) the permanent electric dipole-moment of BaTiO_3_^54^ is much larger than that of BaZrO_3_^55^, and (d) the success of the Mason theory describing the critical-relaxation of normal-ferroelectrics^56,57^, here, the proposed theory of relaxor-ferroelectricity includes the 3-dimensional-extended-random-site-Ising-model (3D-ERSIM) along with the Glauber-dynamics of PSs on a simple cubic lattice (lattice constant *a*_0_), referred to as 3D-ERSIGM, and the model Hamiltonian of RFEs is (SI 2),

1a $$H=-\,J\mathop{\sum }\limits_{k\ne l}^{\{nn\}}\,{{\rm{\sigma }}}_{{k}}{{\rm{\sigma }}}_{{l}}{r}_{k}^{\phi }{r}_{l}^{\phi }-\mathop{\sum }\limits_{k=1}^{N}\,({S}_{k}+{E}_{k})\,{{\rm{\sigma }}}_{{k}}{r}_{k}^{\phi }$$

where, in the right side of Eq. 1a, the 1^st^-term is the Hamiltonian of the 3-dimensional-random-site-Ising-model (3D-RSIM)^25–28^, which is a special form of the random interaction Hamiltonian (Eq. 1 of Kleemann *et al*.^4^); *J* the interaction energy constant between the nearest-neighbor PS pairs; *σ*_*k*_ the k^th^-PS on the lattice, and its two states are represented by *σ*_*k*_= ±1; *ϕ* the concentration of PS-vacancies or 1 − *ϕ* the concentration of PSs on the lattice (*ϕ* = 1/3 for PMN and *ϕ* = *x* for BZ_x_T_1−x_); ${r}_{k}^{\phi }$ the random function that ${r}_{k}^{\phi }=0$ for $r < \phi$, ${r}_{k}^{\phi }=1$ for $r\ge \phi$, and *r* is a randomly generated number between 0 and 1; {*nn*} represents the summation of all the nearest-neighbor PS pairs; the 2^nd^- and 3^rd^-terms are the Hamiltonians related to the RISF and RIEF that originate from the differences in the size and charge of the disorder ions in RFEs, respectively. *S*_*k*_ and *E*_*k*_ are the random effective Zeeman fields related to the RISF and RIEF^4^. Obviously, *E*_*k*_ = 0 in the isovalent RFEs (such as BZ_x_T_1−x_), but ${E}_{k}\ne 0$ in the heterovalent ones (PMN and Sr_x_Ba_1−x_Nb_2_O_6_); and *N* the total number of the unit cells in an RFE. Considering the fact that: (i) Heterovalent and isovalent RFEs have the same characteristics of relaxor-ferroelectricity^31–36,39–41,48–51^, so the influence of *E*_*k*_ to relaxor-ferroelectricity is the secondary compared with the primary 3D-RSIM as pointed by Kleemann *et al*.^4^; and (ii) The similarity of RISF to RIEF^4^, we will use an approximate method to describe their influence (Sec. New mean-field...), and the specific forms of *S*_*k*_ and *E*_*k*_ are not given here.

Moreover, in order to describe the dynamic parameters of the 3D-ERSIM (such as complex-permittivity), here we use the Glauber-dynamics^29,30^, i.e. the transition probability [*w*(σ_*k*_)] from σ_*k*_ to −σ_*k*_ in unit time [Appendix (App.) A of SI] is,

1b $$w({\sigma }_{k})=\frac{\nu }{2}\left[1,-,{\sigma }_{k},\,,\tanh ,(\frac{{F}_{k}}{{k}_{B}T})\right]$$

where ${F}_{k}\equiv J\mathop{\sum }\limits_{l}^{\{nnk\}}{{\rm{\sigma }}}_{{l}}{r}_{l}^{{\phi }}+{S}_{k}+{E}_{k}$ is the local field of *σ*_*k*_, {*nnk*} labels the summation of all the nearest-neighbors of ${\sigma }_{k};\,\nu \equiv {\nu }_{0}\,\exp \left(-,\frac{{U}_{B}}{{k}_{B}T}\right)$, *U*_*B*_ is the energy barrier that PSs stride over during the transition from σ_*k*_ to −σ_*k*_, and *v*_0_ is the orientation vibration frequency of PSs in their local energy valleys; and *k*_*B*_ is the Boltzmann constant.

The main reasons for choosing Glauber-dynamics are that: (a) It satisfies the detailed balance condition; (b) The Weiss, i.e. the single-PS, mean-field form of the 3D-RSIM^58,59^ along with the Glauber-dynamics (3D-RSIGM) when *ϕ* = 0 is the same as the Mason theory that describes the critical-relaxation of 2^nd^-order phase-transition^56,57^; and (c) The corresponding relaxation time predicted by our new mean-field to solve 3D-RSIGM for *ϕ* = 0 is consistent with experimental results^57^ (Sec. Complex-permittivity…).

### New mean-field to solve 3D-ERSIGM

Considering (see SI 3 in details): (a) The existing problems of the approximate theoretical methods for 3D-RSIM^60–62^, and especially there is not any feasible method for solving 3D-RSIGM according to the authors’ knowledge; (b) The successes of the existing multi-spin mean-field methods for solving Ising-model, and particularly, with increasing the spin number that the mean-fields contain, the corresponding results tend to the exact solution of 2D-Ising-model^63–66^; and (c) Inspired by the exact solution of the complex-permittivity of 1D-RSIGM^30^, we propose here a new mean-field of PS-strings (PSSs) that contains more PSs and takes into account the correlation between PSs in 3D-space, referred as PSS-MF, to solve the 3D-ERSIGM. The PSS-MF includes the following four steps:

#### Step-1: PSS construction

**Figure 1:**
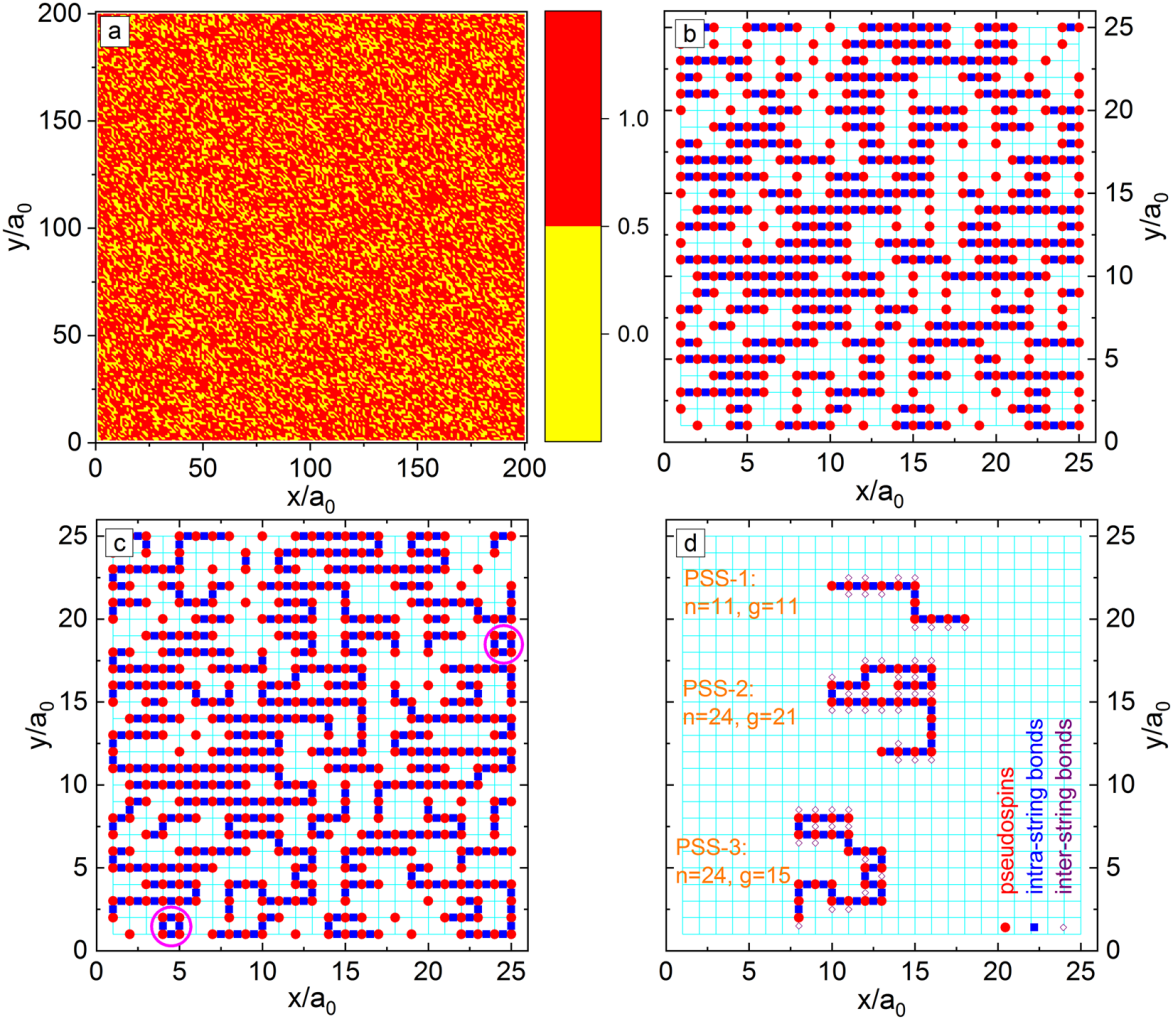
(**a**) Surface plot of the simulated spatial distributions of PSs (red of height 1) and PS-vacancies (yellow of height 0) in the x-y-plane ($z/{a}_{0}=100$) of 3D-RSIM for *ϕ* $=\,1/3$ (a 200 × 200 × 200${{a}_{0}}^{3}$ lattice). (**b**) Simulated PS distribution and connected short PSSs along the x-axis direction in the x-y-plane ($z/{a}_{0}=10$) of 3D-RSIM for *ϕ* $=\,1/3$ (a small 25 × 25 × 25${{a}_{0}}^{3}$ lattice). Cyan lines and red solid circles show the crystal lattice and PSs, respectively, while the unlabeled lattice points are PS-vacancies. A blue solid square indicates that its nearest-neighbor two PSs belong to the same string; (**c**) Connected long PSSs in the x-y plane. They are ring PSSs in the circles of (**c**); and (**d**) Three PSSs selected from (**c**). The red circles, blue squares, and violet diamonds show the PSs, the intra-string and inter-string interaction bonds. PSS-1: $n=11$ and $g=11$. PSS-2: $n=24$ and $g=21$. PSS-3: $n=24$ and $g=15$.

There are six kinds of scans, i.e. x-y-z-, y-x-z-, x-z-y-, z-x-y-, y-z-x-, and z-y-x-scans, to construct PSSs for the 3D-RSIM (Fig. 1a–c). For example, the x-y-z-scan is as the follows: (i) Along the x-axis direction of the crystal lattice, connect the nearest-neighbor PSs into short PSSs (Fig. 1b); (ii) Along the y-axis direction, any two nearest-neighbor endpoints of the short PSSs are connected in the x-y plane to form long PSSs (An endpoint already connected to another PSS is no longer reconnected) as indicated in Fig. 1c; and (iii) Along the z-axis direction, continue to connect any two nearest-neighbor endpoints to form longer PSSs in the 3D-lattice. Here, a PSS containing *n* PSs is expressed as an n-PSS, and the corresponding intra-string Hamiltonian (${H}_{{intra}}^{ng}$) is ${H}_{{intra}}^{ng}=-\,J\mathop{\sum }\limits_{i=1}^{n-1}\,{{\rm{\sigma }}}_{{i}}^{{s}}{{\rm{\sigma }}}_{{i}+1}^{{s}}$, where ${{\rm{\sigma }}}_{{i}}^{{s}}$ indicates the i^th^-PS in the n-PSS, i.e. renumbering the PSs in the 3D-ERSIM Hamiltonian of RFEs (Eq. 1a).

#### Step-2: Calculation of PSS length distribution

Count the number of n-PSSs in the model (Fig. 1c), obtaining the PSS length distribution function (*q*_*n*_) versus (vs) *n*. Due to the spatial isotropy of the 3D-RSIM in Eq. 1a, the calculated *q*_*n*_ is the same for the six scans.

#### Step-3: Calculation of inter-PSS interaction distribution

Count the number of the n-PSSs with the nearest-neighbor number of PSs being *g* (n-g-PSSs) in the model (Fig. 1d), getting the inter-string interaction (bond) distribution function (${\rho }_{{n}}^{{g}}$) vs *n* and *g*.

#### Step-4: Mean-field of PSSs (App. B, Figs.B1 and B2 of SI for details)

The inter-string interaction of an n-g-PSS with its nearest-neighbor PSs, the RISF and RIEF is described by the Weiss-type mean-field^58,59,64^, and the corresponding Hamiltonian (${H}_{inter}^{ng}$) is ${H}_{inter}^{ng}=-\,J\frac{g}{n}\left[\left(1-\frac{1}{{n}^{b}}\right){\eta }_{n}^{g}+\frac{1}{{n}^{b}}\right]\,\mathop{\sum }\limits_{i=1}^{n}\,{{\rm{\sigma }}}_{{i}}^{{s}}$, where *b* is the factor of the effective interface-effect (due to the interfaces between PS and PS-vacancy groups, the RISF and RIEF), and,

2a $${\eta }_{n}^{g}\equiv \frac{1}{n}\mathop{\sum }\limits_{i=1}^{n}{s}_{ni}^{g}$$

In which, ${s}_{ni}^{g}$is the expectation value of ${{\rm{\sigma }}}_{i}^{s}$ (App. C and D of SI).

Therefore, the total Hamiltonian of n-g-PSSs is,

2b $${H}_{n}^{g}=-\,J\mathop{\sum }\limits_{i=1}^{n-1}{\sigma }_{i}^{s}{\sigma }_{i+1}^{s}-J\frac{g}{n}\left[\left(1,-,\frac{1}{{n}^{b}}\right),{\eta }_{n}^{g},+,\frac{1}{{n}^{b}}\right]\,\mathop{\sum }\limits_{j=1}^{n}\,{\sigma }_{j}^{s}$$

and the PSS-MF simplifies the 3D-ERSIM (Eq. 1a) as the following Hamiltonian approximately,

2c $$\begin{array}{rcl}H & \approx  & -N(1-\phi )J\mathop{\sum }\limits_{n=1}^{\infty }\mathop{\sum }\limits_{g=0}^{4n}\,n{q}_{n}{{\rm{\rho }}}_{{n}}^{{g}}\\  & \times  & \left\{\mathop{\sum \,}\limits_{i=1}^{n-1}{{\rm{\sigma }}}_{{i}}^{{s}}{{\rm{\sigma }}}_{{i}+1}^{{s}}+\frac{g}{2n}\left[\left(1-\frac{1}{{n}^{b}}\right){\eta }_{n}^{g}+\frac{1}{{n}^{b}}\right]\,\mathop{\sum }\limits_{j=1}^{n}\,{{\rm{\sigma }}}_{{j}}^{{s}}\right\}\end{array}$$

The normalized condition of *q*_*n*_ and ${{\rm{\rho }}}_{{n}}^{{g}}$ used here are, respectively, $\mathop{\sum }\limits_{n=1}^{\infty }n{q}_{n}=1$ and $\mathop{\sum \,}\limits_{g=0}^{4n}{{\rm{\rho }}}_{{n}}^{{g}}=1$ in this paper.

From ${H}_{n}^{g}$ (Eq. 2b), the Glauber transition probability [${w}_{n}({\sigma }_{i})$] of n-g-PSSs is (App. A of SI),

2d $${w}_{n}({\sigma }_{i}^{s})=\frac{\nu }{2}[1-\gamma {\sigma }_{i}^{s}+{\beta }_{i}(\gamma -{\sigma }_{i}^{s})({\sigma }_{i-1}^{s}+{\sigma }_{i+1}^{s})]$$

where $i=1,\cdots n$, ${{\rm{\sigma }}}_{0}^{{s}}={{\rm{\sigma }}}_{{n}+1}^{{s}}=0$, ${\beta }_{i}\equiv \frac{1}{|{{\rm{\sigma }}}_{{i}-1}^{s}|+|{{\rm{\sigma }}}_{{i}+1}^{{s}}|}\,\tanh [(|{{\rm{\sigma }}}_{{i}-1}^{{s}}|+|{{\rm{\sigma }}}_{{i}+1}^{{s}}|)\varpi ]$,$\,|{{\rm{\sigma }}}_{{i}}^{{s}}|$ is the absolute of ${{\rm{\sigma }}}_{{i}}^{{s}}$, ${\rm{\varpi }}\equiv \frac{{\Theta }_{{J}}}{T}$, ${\Theta }_{{J}}\equiv \frac{J}{{k}_{B}}$, $\gamma \,\equiv \,\tanh (\theta )$, $\theta \equiv \frac{{\Theta }_{n}^{g}}{T}\left[(1-\frac{1}{{n}^{b}}),{\eta }_{n}^{g},+,\frac{1}{{n}^{b}}\right]$, and ${\Theta }_{n}^{g}\equiv \frac{g}{n}{\Theta }_{J}$.

In this article, we use the following computer simulations to show the spatial distribution of PSs and PS-vacancies, and calculate *q*_*n*_ and ${{\rm{\rho }}}_{{n}}^{{g}}$. Specifically: (i) Construct an ensemble which includes 10^4^ 3D-simple-cubic-lattices with 200 × 200 × 200 grid points; and (ii) For any lattice point, a random number *r* between 0 and 1 is first generated by the Intel-Visual-Fortran 2013 program, and there is a PS-vacancy if *r* < *ϕ* or a PS if *r* ≥ *ϕ* on the point, respectively.

### Spatial distribution of PSs and PS-vacancies

Figure 1a shows the distribution of PSs and PS-vacancies in the x-y-plane of a simulated crystal lattice of 3D-RSIM for *ϕ *= 1/3. Due to the randomness of the PSs and PS-vacancies on the lattice, there are accumulated regions or clusters of PSs or PS-vacancies with random local structures. As shown in the Sec. Local-order-parameter, it is just these PS clusters that lead to the appearance of the PNRs^15,16,44–47^ first proposed by Burns *et al*.^42,43^, although its definition is quite unclear now as pointed out by Cowley *et al*.^16^.

### PSS length distribution

**Figure 2:**
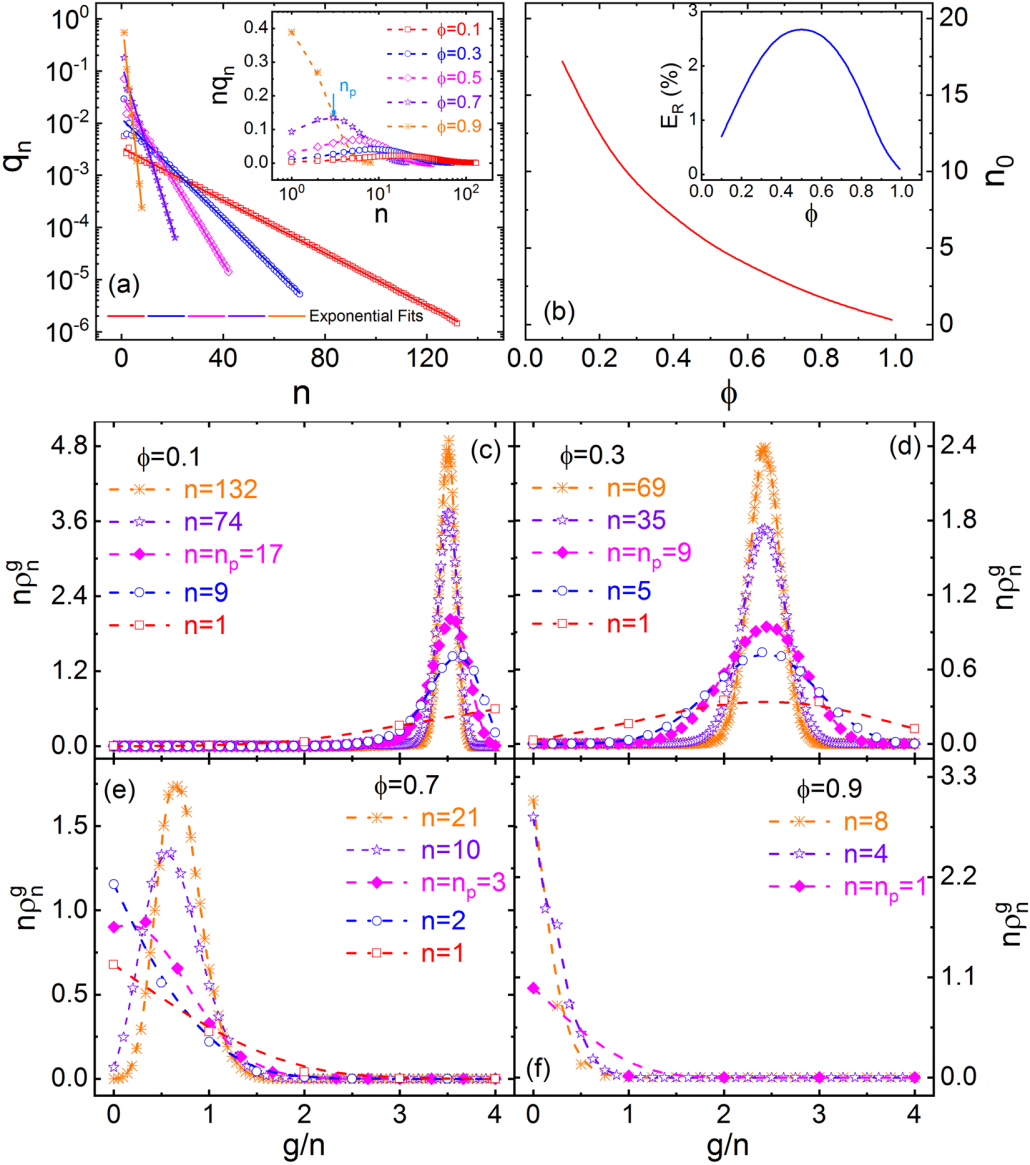
(**a**) *q*_*n*_ vs *n* in 3D-RSIM with $\phi =0.1,\,0.3,\,05,\,07,\,0.9$, and the inset shows $n{q}_{n}$ vs *n* for the corresponding *ϕ* values. ${n}_{p}$ is the peak position of $n{q}_{n}$. (**b**) ${n}_{0}$ vs *ϕ* in 3D-RSIM, and the inset illustrates *E*_*R*_ vs *ϕ*. (**c**–**f**) ${n}_{n}^{g}$ vs $g/n$ and *n* in 3D-RSIM with *ϕ* = 0.1, 0.3, 0.7, and 0.9.

Due to the spatial isotropy of the 3D-ERSIM (Eq. 1a), the obtained *q*_*n*_ and ${{\rm{\rho }}}_{{n}}^{{g}}$ are same by any scan of the six kinds (Sec. New mean-field...). As shown in Fig. 2a, the resulting *q*_*n*_ vs *n* can be described by the following exponential function,

3 $${q}_{n}={q}_{0}{e}^{-n/{n}_{0}}$$

where *n*_0_ is the average length of PSSs, and *q*_0_ is the normalized constant.

The probability that a PS belongs to an n-PSS is *nq*_*n*_, and *nq*_*n*_ vs *n* is given in the inset of Fig. 2a. *nq*_*n*_ appears as a single peak with *n*, and the corresponding *n* value (*n*_*p*_) of the peak position decreases until *n*_*p*_ = 1 as *ϕ* increases (Table S4.1 of SI 4).

The calculated *n*_0_ vs *ϕ* is illustrated in Fig. 2b, and *n*_0_ decreases with increasing *ϕ*. As shown in Fig. 1, *n* can be used as a size measure of the PS clusters in 3D-RSIM, while *n*_0_ is their characteristic size. In this paper, the ring PSSs in 3D-RSIM are ignored (Fig. 1c). The probability (*E*_*R*_) that the PSs belong to the ring PSSs is equal to the number of all PSs in the rings divided by the total number of PSs in the model. *E*_*R*_ vs *ϕ* is shown in the inset of Fig. 2b, and the maximum *E*_*R*_ is about 2.7%.

### Inter-PSS interaction distribution

The simulated ${{\rm{\rho }}}_{{n}}^{{g}}$ vs *g* of 3D-RSIM for serial *ϕ* and *n* is presented in Fig. 2c–f. There is a single peak of ${{\rm{\rho }}}_{{n}}^{{g}}$ vs *g* for all *ϕ* and *n*, and it could be imagined that ${{\rm{\rho }}}_{{n}}^{{g}}$ for *ϕ* → 0 is a Dirac δ-function at $\frac{g}{n}=4$. By representing the *g* value corresponding to the maximum of ${{\rm{\rho }}}_{{n}}^{{g}}$ as ${g}_{p}^{n}$, we could see that: (a) There is a threshold at ${\phi }_{{\rm{c}}}\approx 0.3$, where ${g}_{p}^{n}$ is nearly irrelevant to *n* and ${g}_{p}^{n}\approx 2.45$ (Fig. 2d); and (b) ${g}_{p}^{n}$ becomes smaller for *ϕ* < *ϕ*_*c*_ or larger for *ϕ* < *ϕ*_*c*_ as *n* goes up. In other words, although the sizes of PS clusters in 3D-RSIM of *ϕ *≈ *ϕ*_*c*_ are different, the average coordination PS number per PS in the clusters is nearly same, and this number is equal to ${g}_{p}^{n}+2\approx 4.45$. Obviously, this phenomenon is related to the percolation of PS-vacancies if we change the view angle. In fact, *ϕ*_*c*_ is near to the percolation threshold (=0.31) of PS-vacancies in 3D-RSIM^67^, and we consider that they are equal and have the same physical origin in this article. We would like to point out that *ϕ*_*c*_ can be defined as the characteristic concentration of the canonical RFEs (Sec. Definition of canonical RFEs).

Moreover, the ${{\rm{\rho }}}_{{n}}^{{g}}$ vs *g* for *n* = *n*_*p*_ (the magenta diamond symbols in Fig. 2c–f) indicates that there is another threshold at $\phi \approx 0.7$, and this value is near to the percolation threshold (${\phi }_{p}=0.69$) of PSs in 3D-RSIM^67^ (Here, we also consider this threshold is equal to *ϕ*_*p*_). Specifically, (i) when *ϕ* < *ϕ*_*p*_, ${g}_{p}^{n}({n}_{p})=0$, and the ${{\rm{\rho }}}_{n}^{g}$ peak becomes narrow with increasing *ϕ*; and (ii) For *ϕ *< *ϕ*_*p*_, ${g}_{p}^{n}({n}_{p})$ decreases while the ${{\rm{\rho }}}_{{n}}^{{g}}$ peak widens as *ϕ* goes up.

### Order-parameter, static-permittivity and specific-heat

When the n-g-PSSs are in thermal equilibrium, the corresponding equilibrium value (${\eta }_{ne}^{g}$) of ${\eta }_{n}^{g}$, i.e. the order-parameter of the n-g-PSSs, is,

4a $${\eta }_{ne}^{g}={\left[\frac{1}{n{Z}_{n}^{g}}\frac{\partial {Z}_{n}^{g}}{\partial {\rm{\theta }}}\right]}_{{\rm{\theta }}={{\rm{\theta }}}_{{\rm{e}}}}={\left[\gamma +\frac{1-{\gamma }^{2}}{n{Q}_{n}^{g}}\frac{\partial {Q}_{n}^{g}}{\partial \gamma }\right]}_{\gamma ={\gamma }_{{\rm{e}}}}$$

where ${Z}_{n}^{g}$ is the partition function of n-g-PSSs corresponding to ${H}_{n}^{g}$ (Eq. 2b), ${Q}_{n}^{g}$ is an intermediate variable to calculate ${Z}_{n}^{g}$ (App. C of SI), ${\gamma }_{{\rm{e}}}\equiv \,\tanh ({{\rm{\theta }}}_{{\rm{e}}})$, and ${{\rm{\theta }}}_{{\rm{e}}}\equiv \frac{{\Theta }_{{n}}^{{g}}}{T}\left[\left(1-\frac{1}{{n}^{b}}\right){\eta }_{ne}^{g}+\frac{1}{{n}^{b}}\right]$.

From Eq. 4a, the static-permittivity (${{\chi }}_{s}^{ng}$) of the n-g-PSSs in thermal equilibrium is (App. E of SI),

4b $${{\chi }}_{s}^{ng}=\frac{{C}_{w}}{{N}_{0}}\frac{n{\aleph }_{n}^{g}}{T-{\aleph }_{n}^{g}{A}_{n}^{g}}$$

where ${\aleph }_{n}^{g}$ is an intermediate variable (App. E of SI), ${A}_{n}^{g}\equiv \left(1-\frac{1}{{n}^{b}}\right){\Theta }_{{n}}^{{g}}$, ${C}_{w}\equiv \frac{{N}_{0}{\mu }^{2}}{{\varepsilon }_{0}{k}_{B}}$ the Curie-Weiss constant, *N*_0_ the number of the lattice points per unit volume, and *ε*_0_ the vacuum dielectric constant.

By ${H}_{n}^{g}$ (Eq. 2b), the average internal energy (${u}_{n}^{g}$) and specific-heat (${c}_{n}^{g}$) per PS of the n-g-PSSs in thermal equilibrium are,

4c $${u}_{n}^{g}=-\,\frac{J}{n}\mathop{\sum }\limits_{i=1}^{n-1}{\zeta }_{ni}^{ge}-\frac{J}{2}\frac{g}{n}\left[\left(1-\frac{1}{{n}^{b}}\right){\eta }_{ne}^{g}+\frac{1}{{n}^{b}}\right]{\eta }_{ne}^{g}$$

4d $${c}_{n}^{g}=\frac{\partial {u}_{n}^{g}}{\partial T}$$

where ${\zeta }_{ni}^{ge}$ is the equilibrium value of the correlation-function (${\zeta }_{ni}^{g}$) between the i^th^- and (i + 1)^th^-PSs, i.e. the expectation value of ${{\rm{\sigma }}}_{{i}}^{{s}}{{\rm{\sigma }}}_{{i}+1}^{{s}}$, in the n-g-PSSs (App. F of SI).

**Figure 3:**
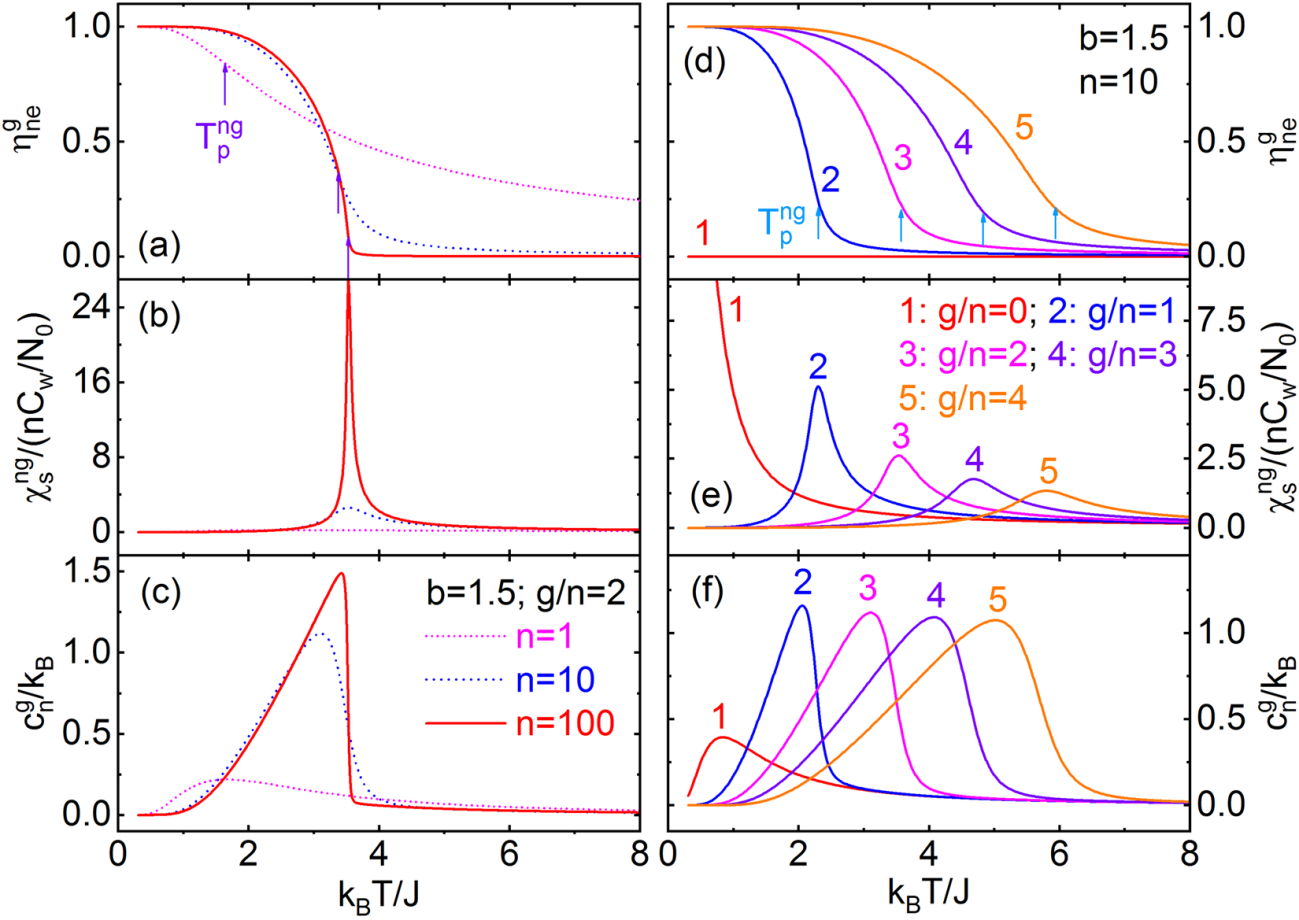
(**a**) ${\eta }_{ne}^{g}$, (**b**) ${{\chi }}_{s}^{ng}$, and (**c**) ${c}_{n}^{g}$ of the n-g-PSSs vs *T* for $b=1.5$, $g/n=2$, $n=1$ (pink dot line), 10 (blue dash), and 100 (red solid). (**d**) ${\eta }_{ne}^{g}$, (**e**) ${{\chi }}_{s}^{ng}$, and (**f**) ${c}_{n}^{g}$ of the n-g-PSSs vs *T* for $b=1.5$,$\,n=10$, $g/n=0$ (red solid line #1), 1 (blue #2), 2 (pink #3), 3 (violet #4), and 4 (orange #5). ${T}_{p}^{ng}$ in (**a**,**d**) is the DPT temperature.

Figure 3 shows the ${\eta }_{ne}^{g}$, ${{\chi }}_{s}^{\,ng}$, and ${c}_{n}^{g}$ vs *T* for *b* = 1.5, serial *n* and *g*. We obtain that, with decreasing *n* and for non-zero *g*, n-g-PSSs have an evolution from 2^nd^-order phase-transition to DPT, which is indicated by the diffuse change of ${\eta }_{ne}^{g}$ as well as the dispersion peaks of ${{\chi }}_{s}^{\,ng}$ and ${c}_{n}^{g}$, and the DPT spreads to a wider temperature zone. In this article, we define the temperature corresponding to the maximum value of $-\frac{{\rm{\partial }}{\eta }_{ne}^{g}}{{\rm{\partial }}T}$ as the transition temperature (${T}_{p}^{ng}$) of the DPT of n-g-PSSs, and ${T}_{p}^{ng}$ goes up with increasing *n* and *g*. ${T}_{p}^{ng}\equiv 0$ for n-g-PSSs with *g* = 0 (n-0-PSSs), giving that they belong to the paraelectric-subsystem in 3D-ERSIM. n-0-PSSs has a small diffuse ${c}_{n}^{g}$ peak at low-temperature except *n* = 1 (Fig. 3f). Physically, for finite *n* and *g* > 0, the non-zero ${\eta }_{ne}^{g}$ value at temperature higher than ${T}_{p}^{ng}$ originates from that the existence of the PS-vacancy groups, RISF and RIEF makes the probability of the ferroelectric configurations in 3D-ERSIM being higher compared with 3D-Ising-model at high temperature^58,59,64^ (App. B of SI).

The order-parameter ($\eta$), spontaneous-polarization (*P*_*s*_), static-permittivity (${{\chi }}_{s}^{\,ps}$), as well as the average internal energy (*u*_*ps*_) and average specific-heat (*c*_*ps*_) per PS of 3D-ERSIM are,

5a $$\eta =(1-\phi )\mathop{\sum }\limits_{n=1}^{\infty }\mathop{\sum }\limits_{g=0}^{4n}n{q}_{n}{{\rm{\rho }}}_{{n}}^{{g}}{\eta }_{{ne}}^{{g}}$$

5b $${P}_{s}\approx {N}_{0}\mu \eta $$

5c $${{\chi }}_{s}^{ps}\approx {N}_{0}(1-\phi )\mathop{\sum }\limits_{n=1}^{\infty }\,\mathop{\sum }\limits_{g=0}^{4n}\,{q}_{n}{{\rm{\rho }}}_{{n}}^{{g}}{{\chi }}_{s}^{ng}$$

5d $${u}_{ps}=(1-\phi )\mathop{\sum }\limits_{n=1}^{\infty }\,\mathop{\sum }\limits_{g=0}^{4n}\,n{q}_{n}{{\rm{\rho }}}_{{n}}^{{g}}{u}_{n}^{g}$$

5e $${c}_{ps}=(1-\phi )\mathop{\sum }\limits_{n=1}^{\infty }\mathop{\sum }\limits_{g=0}^{4n}\,n{q}_{n}{{\rm{\rho }}}_{{n}}^{{g}}{c}_{n}^{g}$$

In the calculation of macroscopic *P*_*s*_ and ${{\chi }}_{s}^{\,ps}$ by the microscopic ${\eta }_{ne}^{g}$ and ${{\chi }}_{s}^{\,ng}$, this paper uses an approximation similar to the parallel capacitance circuits (Eq. 5b,c).

**Figure 4:**
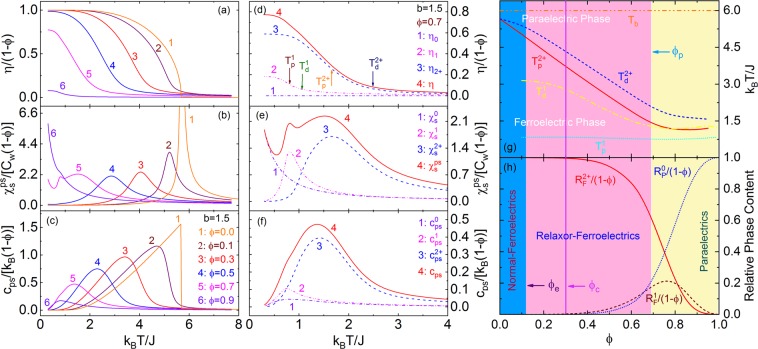
(**a**–**c**) $\eta$, ${{\chi }}_{s}^{\,ps}$, and ${c}_{ps}$ of 3D-ERSIM with $\phi =0,\,0.1,\,0.3,\,0.5,\,0.7,\,0.9$ vs $T$ for $b=1.5$. (**d**–**f**) Order-parameter, static-permittivity, and average specific-heat per PS of 3D-ERSIM, n-0-PSSs, n-1-PSSs, and n-2^+^-PSSs in the model vs $T$ for *ϕ* = 0.7 and $b=1.5$. ${T}_{p}^{1}$ and ${T}_{p}^{2+}$ are the transition temperatures, as well as ${T}_{d}^{1}$ and ${T}_{d}^{2+}$ the diffuse temperatures of n-1-PSSs and n-2^+^-PSSs. (**g**,**h**) Phase diagram of 3D-ERSIM with $b=1.5$, i.e. ${T}_{p}^{1}$, ${T}_{p}^{2+}$, ${T}_{d}^{1}$, ${T}_{d}^{2+}$, ${R}_{P}^{0}$, ${R}_{F}^{1}$, ${R}_{F}^{2+}$, and ${T}_{b}$ vs *ϕ*. *ϕ*_*e*_ and *ϕ*_*p*_ (PS percolation threshold of 3D-RSIM) are, respectively, the characteristic concentrations of the evolutions between the normal-ferroelectrics $\leftrightarrow$ RFEs $\leftrightarrow$ paraelectrics. *ϕ*_*c*_ (PS-vacancy percolation threshold of 3D-RSIM) is the characteristic concentration of the canonical RFEs (Sec. Inter-PSS interaction distribution and Sec. Definition of canonical RFEs).

For serial *ϕ* and $b=1.5$, *η*, ${{\chi }}_{s}^{ps}$, and ${c}_{ps}$ vs *T* are shown in Fig. 4a–c, and it could be seen that: (i) With varying *ϕ*, 3D-ERSIM has the evolutions between the normal-ferroelectrics of (nearly) 2^nd^-order phase-transition ↔ RFEs of DPT ↔ paraelectrics of (almost) no PS ordering as indicated by the very small *η* for $\phi =0.9$ and rapid increase of ${{\chi }}_{s}^{\,ps}$ with decreasing *T* (Fig. 4a,b); (ii) As *ϕ* increases, $\frac{\eta (T\to 0)}{1-\phi }$ decreases (Fig. 4a), indicating that only part of the PSs become ferroelectric, i.e. ferroelectric- and paraelectric-subsystems coexist in 3D-ERSIM; and (iii) There are a low-temperature- and a high-temperature-DPTs corresponding to the two peaks of ${{\chi }}_{s}^{\,ps}$ (the line 5 in Fig. 4b) for $\phi \approx {\phi }_{p}$.

Detailed analyses (Fig. 4d–f) show that the paraelectric-subsystem is just the n-0-PSSs in 3D-ERSIM, and its content (${R}_{P}^{0}$), order-parameter (${\eta }_{0}$), static-permittivity (${{\chi }}_{s}^{\,0}$), and specific-heat (${c}_{ps}^{0}$) per PS are,

6a $${R}_{P}^{0}=(1-\phi )\mathop{\sum }\limits_{n=1}^{\infty }n{q}_{n}{{\rm{\rho }}}_{{n}}^{0}$$

6b $${\eta }_{0}\equiv 0$$

6c $${\chi }_{s}^{0}\approx {N}_{0}(1-\phi )\mathop{\sum }\limits_{n=1}^{{\rm{\infty }}}\,{q}_{n}{\rho }_{n}^{0}{\chi }_{s}^{n0}$$

6d $${c}_{ps}^{0}=(1-\phi )\mathop{\sum }\limits_{n=1}^{\infty }\,n{q}_{n}{{\rm{\rho }}}_{n}^{0}{c}_{n}^{0}$$

The low-temperature- and high-temperature-DPTs correspond to n-g-PSSs with *g* = 1 (n-1-PSSs) and n-g-PSSs of *g* ≥ 2 (n-2^+^-PSSs), respectively. The content (${R}_{F}^{1}$), order-parameter (*η*_1_), spontaneous-polarization (${P}_{s}^{1}$), static-permittivity (${{\chi }}_{s}^{\,1}$), and average specific-heat per PS (${c}_{ps}^{1}$) of n-1-PSSs are,

7a $${R}_{F}^{1}=(1-\phi )\mathop{\sum }\limits_{n=1}^{\infty }n{q}_{n}{{\rm{\rho }}}_{{n}}^{1}$$

7b $${\eta }_{1}=(1-\phi )\mathop{\sum }\limits_{n=1}^{\infty }n{q}_{n}{{\rm{\rho }}}_{{n}}^{1}{\eta }_{ne}^{1}$$

7c $${P}_{s}^{1}\approx {N}_{0}\mu {\eta }_{1}$$

7d $${{\chi }}_{s}^{1}\approx {N}_{0}(1-\phi )\mathop{\sum }\limits_{n=1}^{\infty }{q}_{n}{{\rm{\rho }}}_{{n}}^{1}{{\chi }}_{s}^{n1}$$

7e $${c}_{ps}^{1}=(1-\phi )\mathop{\sum }\limits_{n=1}^{\infty }n{q}_{n}{{\rm{\rho }}}_{{n}}^{1}{c}_{n}^{1}$$

The content (${R}_{F}^{2+}$), order-parameter (${\eta }_{2+}$), spontaneous-polarization (${P}_{s}^{2+}$), static-permittivity (${{\chi }}_{s}^{\,2+}$), and average specific-heat per PS (${c}_{ps}^{2+}$) of n-2^+^-PSSs are,

8a $${R}_{F}^{2+}=(1-\phi )\mathop{\sum }\limits_{n=1}^{\infty }\mathop{\sum }\limits_{g=2}^{4n}n{q}_{n}{{\rm{\rho }}}_{{n}}^{{g}}$$

8b $${\eta }_{2+}=(1-\phi )\mathop{\sum }\limits_{n=1}^{\infty }\,\mathop{\sum }\limits_{g=2}^{4n}\,n{q}_{n}{{\rm{\rho }}}_{n}^{g}{\eta }_{ne}^{g}$$

8c $${P}_{s}^{2+}\approx {N}_{0}\mu {\eta }_{2+}$$

8d $${{\chi }}_{s}^{2+}\approx {N}_{0}(1-\phi )\mathop{\sum }\limits_{n=1}^{\infty }\,\mathop{\sum }\limits_{g=2}^{4n}\,{q}_{n}{{\rm{\rho }}}_{{n}}^{{g}}{{\chi }}_{s}^{ng}$$

8e $${c}_{ps}^{2+}=(1-\phi )\mathop{\sum }\limits_{n=1}^{\infty }\mathop{\sum }\limits_{g=2}^{4n}\,n{q}_{n}{{\rm{\rho }}}_{{n}}^{{g}}{c}_{n}^{g}$$

In order to quantitatively describe the high-temperature- and low-temperature-DPTs of 3D-ERSIM, this paper defines that: (i) The temperatures corresponding to the maximum values of $-\frac{d{\eta }_{1}}{dT}$ and $-\frac{d{\eta }_{2+}}{dT}$ are the phase-transition temperatures of n-1-PSSs (${T}_{P}^{1}$) and n-2^+^-PSSs (${T}_{P}^{2+}$); and (ii) To show the dispersion of the DPTs, the corresponding diffuse temperatures (${T}_{d}^{1}$ and ${T}_{d}^{2+}$) are determined by $\frac{{\eta }_{1}({T}_{d}^{1})}{{\eta }_{1}({T}_{P}^{1})}\equiv \frac{1}{e}$ and $\frac{{\eta }_{2+}({T}_{d}^{2+})}{{\eta }_{2+}({T}_{P}^{2+})}\equiv \frac{1}{e}$ ($e\equiv 2.718\cdots$, i.e. the Euler or natural number), as shown in Fig. 4d.

The phase diagram of 3D-ERSIM with *b* = 1.5 is shown in Fig. 4g–h, i.e. ${T}_{p}^{1}$, ${T}_{p}^{2+}$, ${T}_{d}^{1}$, ${T}_{d}^{2+}$, ${R}_{P}^{0}$, ${R}_{F}^{1}$, ${R}_{F}^{2+}$, and ${T}_{b}$ (Burns temperature^15,16^, see Sec. Burns-transformation) vs *ϕ*, which indicates that, as *ϕ* increases: (i) ${T}_{p}^{2+}$ first decreases, but it remains almost unchanged after $\phi \approx 0.8$; ${T}_{d}^{2+}$ first drops slightly, then rapidly, and keeps as a constant after $\phi \approx 0.8$; ${T}_{d}^{1}$ first decreases slowly, then rapidly, and increases slightly at the end; as well as ${T}_{p}^{2+}$ is always higher than ${T}_{p}^{1}$, and$\,{T}_{p}^{1}$ is almost irrelevant to *ϕ*; (ii) ${R}_{P}^{0}$ and ${R}_{F}^{2+}$, respectively, increases and decreases monotonically, with the maximum growth or drop rate near $\phi =0.8$; ${R}_{F}^{1}$ shows a diffuse peak with the peak position near $\phi =0.75$. In other words, 3D-ERSIM has three subsystems: paraelectric- (n-0-PSSs), low-transition-temperature-ferroelectric- (n-1-PSSs), and high-transition-temperature-ferroelectric- (n-2^+^-PSSs) subsystems. The dominant subsystem is n-2^+^-PSSs when *ϕ* is small; n-2^+^-PSSs, n-1-PSSs, and n-0-PSSs almost have the same contents near $\phi ={\phi }_{p}$; and n-0-PSSs dominates the whole system when *ϕ* is large enough; and (iii) n-2^+^-PSSs gradually evolve from the normal-ferroelectrics of (nearly) 2^nd^-order phase-transition to RFEs of DPT, and the characteristic concentration (*ϕ*_*e*_) of PS-vacancies of this evolution is about 0.12 [defined here by the crossover from the slight to rapid drops of ${T}_{d}^{2+}$ (Fig. 4g)], which stems from the decrease of characteristic PS cluster size (*n*_0_) as shown in Fig. 2b and distribution broadening of the interaction between the clusters (Fig. 2c,d). Compared with n-2^+^-PSSs, n-1-PSSs always have narrow DPT because they have no interaction distribution.

### Local-order-parameter

When the n-g-PSSs are in thermal equilibrium, the corresponding equilibrium value (${s}_{ni}^{ge}$) of ${s}_{ni}^{g}$ (App. D of SI) is,

9 $${s}_{ni}^{ge}={\left[\frac{1}{{Q}_{n}^{g}}({Y}_{i}^{g}{Q}_{n-i}^{g}+\alpha {Q}_{i}^{g}{Y}_{n-i}^{g})\right]}_{\gamma ={\gamma }_{{\rm{e}}}}$$

**Figure 5:**
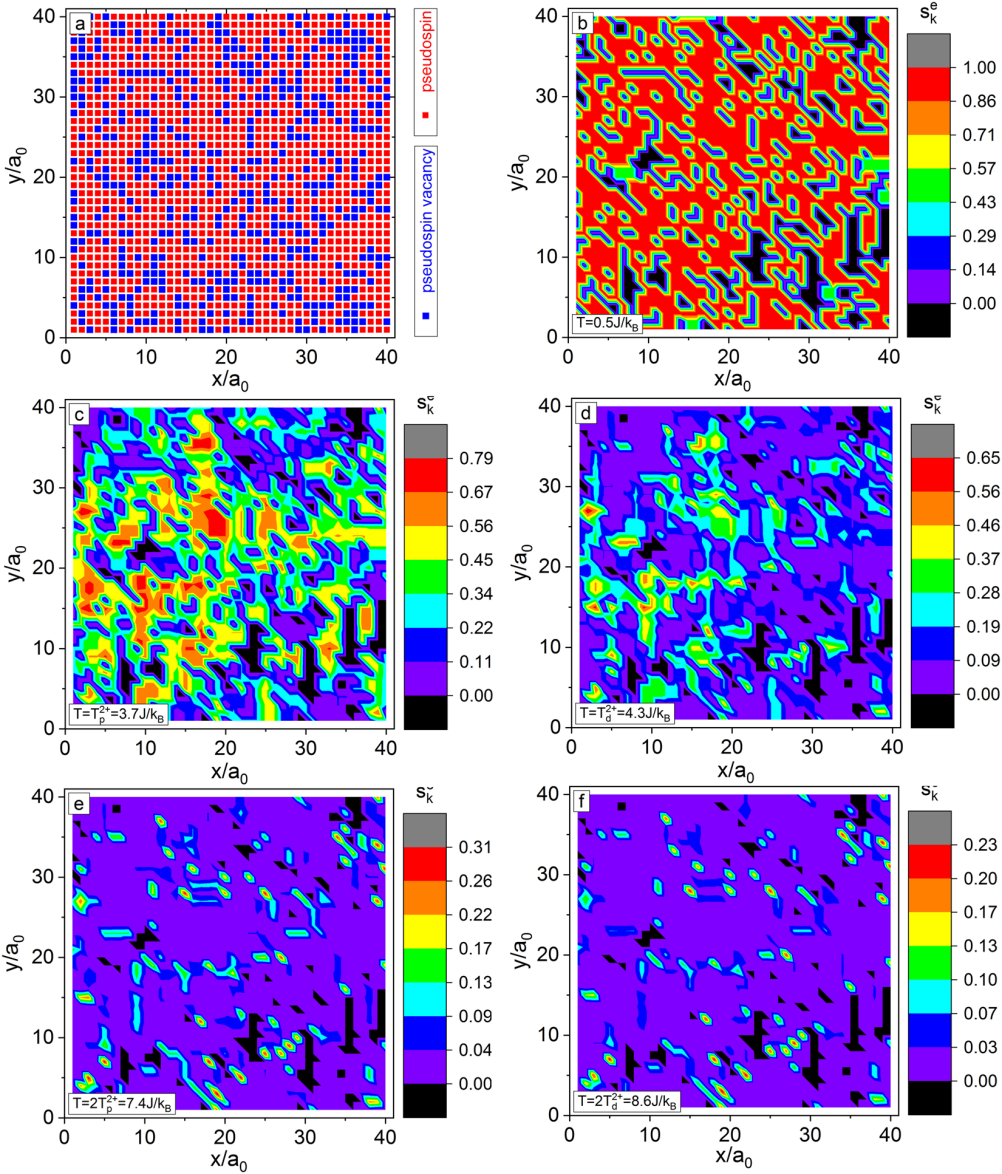
(**a**) Simulated spatial distributions of PSs and PS-vacancies in the x-y-plane ($z/{a}_{0}=20$) of 3D-ERSIM for $\phi =\,1/3$ and $b=1.5$ (40 × 40 × 40 lattice points). Red and blue squares show the PSs and PS-vacancies, respectively. (**b**–**f**) Surface plots of the calculated ${s}_{k}^{e}$ in the x-y-plane when $T=0.5\,J/{k}_{B}=0.14{T}_{p}^{2+}$ (**b**), $3.7\,J/{k}_{B}={T}_{p}^{2+}$ (**c**), $4.3\,J/{k}_{B}={T}_{d}^{2+}$ (**d**), $7.4\,J/{k}_{B}=2{T}_{p}^{2+}$ (**e**), and $8.6\,J/{k}_{B}=2{T}_{d}^{2+}$ (**f**).

Based on ${s}_{ni}^{ge}$, the equilibrium expectation value (${s}_{k}^{e}$) of σ_*k*_, i.e. the local-order-parameter of 3D-ERSIM (Eq. 1a), can be obtained (The specific calculation method of ${s}_{k}^{e}$ is given in the SI 5). The calculated ${s}_{k}^{e}$ results at serial temperatures in the x-y-plane of 3D-ERSIM for $\phi =1/3$ and $b=1.5$ are shown in Fig. 5. It could be seen that: (i) ${s}_{k}^{e}$ shows significant spatial heterogeneity from 0 K to the temperatures far above ${T}_{p}^{2+}$ (Fig. 5b–f) due to the existence of the PS and PS-vacancy clusters (Fig. 5a); (ii) The black regions in Fig. 5b–f that never polarize correspond to those of PS-vacancies (Fig. 5a); (iii) At very low-temperature ($0.14{T}_{p}^{2+}$), the PS shells adjacent to the PS-vacancy regions have smaller ${s}_{k}^{e}$ compared with those inside the PS clusters (Fig. 5b); (iv) Even at such high-temperature ($2{T}_{d}^{2+}$), individual polarized regions of nanoscale about a few *a*_0_, i.e. PNRs, with small ${s}_{k}^{e}$ appear in the PS clusters (Fig. 5f), which originates from the effective interface-effect of PS clusters (App. B of SI); and (v) With decreasing *T*, the size of the PNRs first increases (Fig. 5f,e), then new PNRs appear (Fig. 5e,d), and finally they interconnect to form large polarized regions with quasi-fractal structure (Fig. 5d-b), meanwhile the average of ${s}_{k}^{e}$ always increases.

Along with the DPT, there are at least three different kinds of interfaces in RFEs: (i) *Phase boundary*. The interfaces between adjacent paraelectric and ferroelectric regions (Fig. 5c–f); (ii) *Sub-phase boundary*. The interfaces between adjacent ferroelectric regions of different ${s}_{k}^{e}$ values (Fig. 5b–d); and (iii) *Domain wall*. The interfaces in ferroelectric regions with opposite polarization directions of ${s}_{k}^{e}$. According to the domain formation theory^68–70^, the ferroelectric regions in 3D-ERSIM must become multi-domains due to the influence of the PS-vacancy groups within and adjacent to the PS clusters, the RISF and RIEF.

### Complex-permittivity of correlated-relaxation of PSs

The correlated-relaxation of PSs in normal-ferroelectrics of 2^nd^-order phase-transition is also referred to as the critical-relaxation or phase-transition relaxation because it appears in the vicinity of the critical-temperature^56,57^. To date, the most successful theory of the correlated-relaxation is given by Mason^56^, and it is a mean-field of single PS of 3D-RSIGM for $\phi =0$.

The complex-permittivity (including the linear and higher order) of n-g-PSSs is related to the change of ${s}_{ni}^{g}$ with time (*t*). Based on Eq. 2b,d, we obtain that the equation of ${s}_{ni}^{g}$ vs *t* is (App. G of SI),

10 $$\frac{1}{\nu }\frac{d{s}_{ni}^{g}}{dt}=-\,{s}_{ni}^{g}+{\beta }_{i}({s}_{ni-1}^{g}+{s}_{ni+1}^{g})+\gamma [1-{\beta }_{i}({\zeta }_{ni-1}^{g}+{\zeta }_{ni}^{g})]$$

where $i=1,\cdots \,n$. Due to the interaction between PSs, the evolution of ${s}_{ni}^{g}$ is interrelated to ${\zeta }_{ni}^{g}$ $(i=1,\,\cdots \,n-1)$ (App. F of SI).

The linear complex-permittivity of the correlated-relaxation of n-g-PSSs is directly related to the sufficiently small deviation (${\delta }_{ni}^{g}$) of ${s}_{ni}^{g}$ from its equilibrium value (${s}_{ni}^{ge}\equiv {{s}_{ni}^{g}|}_{\gamma ={\gamma }_{{\rm{e}}}}$), i.e. ${\delta }_{ni}^{g}\equiv {s}_{ni}^{g}-{s}_{ni}^{ge}$. By Eq. 10, we get that (App. H and I of SI),

11 $${{\chi }}_{n}^{{g}^{\ast }}={{\chi }}_{n}^{g{\prime} }-{i}_{c}{{\chi }}_{n}^{g{\prime\prime} }\approx \frac{{{\chi }}_{s}^{\,ng}}{1+{i}_{c}{\omega }{\tau }_{nn}^{g}}$$

where ${i}_{c}$ is the imaginary unit, ω angular frequency, and ${\tau }_{nn}^{g}$ the longest relaxation time of the spatial-relaxation-modes of the correlated-relaxation of n-g-PSSs (Figs. H1, H2 and I1 of App. H and I).

**Figure 6:**
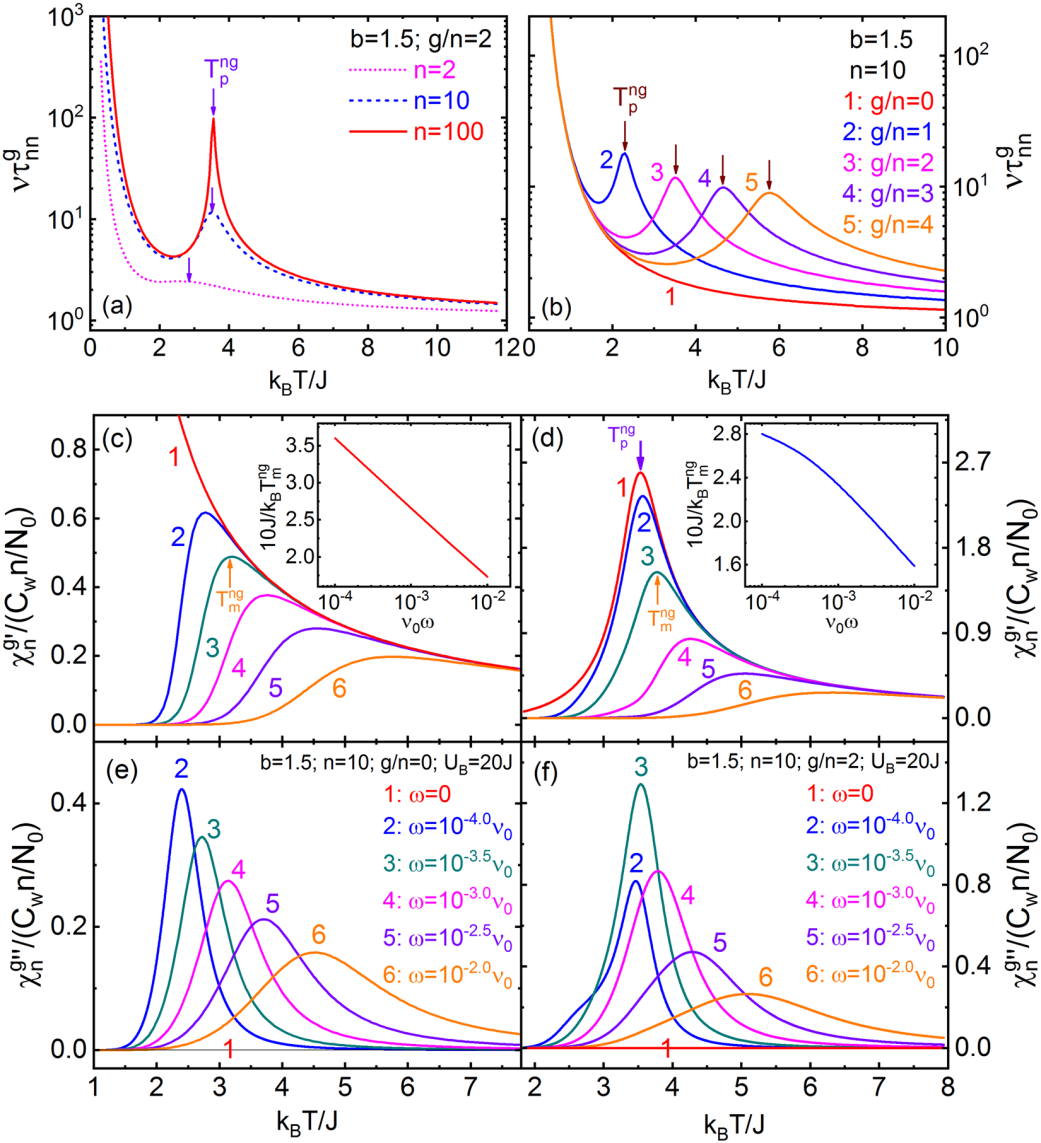
${\rm{\nu }}{\tau }_{nn}^{g}$of n-g-PSSs with $b=1.5$ vs $T$ for $g/n=2$ and $n=2,\,10,\,100$ (**a**), and for $n=10$ and $g/n=0$, 1, 2, 3, 4 (**b**). (**c**–**f**) ${\chi }_{n}^{\,g{\rm{{\prime} }}}$ and ${{\chi }}_{n}^{\,{g}^{{\prime\prime} }}$ of n-g-PSSs with $b=1.5$ and ${U}_{B}=20\,J$ vs *T* and ω for $n=10$ and $g/n=0$ (**c**,**e**), 2 (**d**,**f)**. ${T}_{m}^{ng}$ is the temperature corresponding to the maximum of ${\chi }_{n}^{\,g{\rm{{\prime} }}}$. Insets of (**c**,**d**) show $1/{T}_{m}^{ng}$ vs ω.

Figure 6a,b illustrates that, with decreasing *n* and for nonzero *g*, $\nu {\tau }_{nn}^{g}$ shows a crossover from the λ-shape to diffuse peak near ${T}_{p}^{ng}$, and from the power-law $(\nu {\tau }_{nn}^{g}\sim \frac{1}{T-{T}_{p}^{ng}})$^56,57^ to Vogel-Fulcher-law^31–33^ [${\rm{l}}{\rm{n}}(\nu {\tau }_{nn}^{g})\sim \frac{1}{T-{T}_{v}}$, where *T*_*v*_ is Vogel temperature] above ${T}_{p}^{ng}$ (The relaxation time vs *T* between the power-law and Arrhenius-relation can be described by the Vogel-Fulcher-law approximately), which indicates that the Vogel-Fulcher-law originates from the effective interface-effect of PS clusters (App. B of SI). $\nu {\tau }_{nn}^{g}$ with *g* = 0 always show Arrhenius behavior, and has the same divergent tendency at low-temperature for all *g* values. Obviously, ${{\tau }_{nn}^{4n}|}_{n\to \infty }$ corresponds to the case of normal-ferroelectrics with 2^nd^-order phase-transition (Fig. 6a), and its power-law behavior is consistent with experimental results^57^.

Figure 6c–f give, when $b=1.5$, ${U}_{B}=20\,J$, and $n=10$, ${{\chi }}_{n}^{\,g{\prime} }$ and ${{\chi }}_{n}^{\,{g}^{{\prime\prime} }}$ vs *T*, serial ω and *g* calculated by Eq. 11. It could be seen that: (i) ${{\chi }}_{n}^{\,g{\prime} }$ and ${{\chi }}_{n}^{\,{g}^{{\prime\prime} }}$ have relaxation peaks, and the high-temperature side of ${{\chi }}_{n}^{g{\prime} }$ is almost independent of ω; and (ii) The peak temperature (${T}_{m}^{ng}$) of ${{\chi }}_{n}^{g{\prime} }$ vs ω follows the Arrhenius-relation for *g* = 0 or Vogel-Fulcher-law for $g\ne 0$ (Insets of Fig. 6c,d).

${{\chi }}_{n}^{{g}^{\ast }}$ in 3D-ERSIGM has a distribution with both *n* and *g* (Eqs. 11 and 4b). However, there is still no exact method to calculate the complex-permittivity of a heterogeneous system on molecular scale. Here, an extended-Wagner-approximation^71^ is used to calculate the complex-permittivity (${{\chi }}_{ps}^{\ast }$) of 3D-ERSIGM, i.e.,

12 $${{\chi }}_{ps}^{\ast }={{\chi }}_{ps}^{{\prime} }-{i}_{c}{{\chi }}_{ps}^{{\prime\prime} }\approx {N}_{0}(1-\phi )\mathop{\sum }\limits_{n=1}^{\infty }\,\mathop{\sum }\limits_{g=0}^{4n}\,{q}_{n}{{\rm{\rho }}}_{n}^{g}{{\chi }}_{n}^{\,{g}^{\ast }}$$

where ${{\chi }}_{ps}^{{\prime} }$ and ${{\chi }}_{ps}^{\,{\prime\prime} }$ are the real and imaginary parts of ${{\chi }}_{ps}^{\ast }$, respectively.

**Figure 7:**
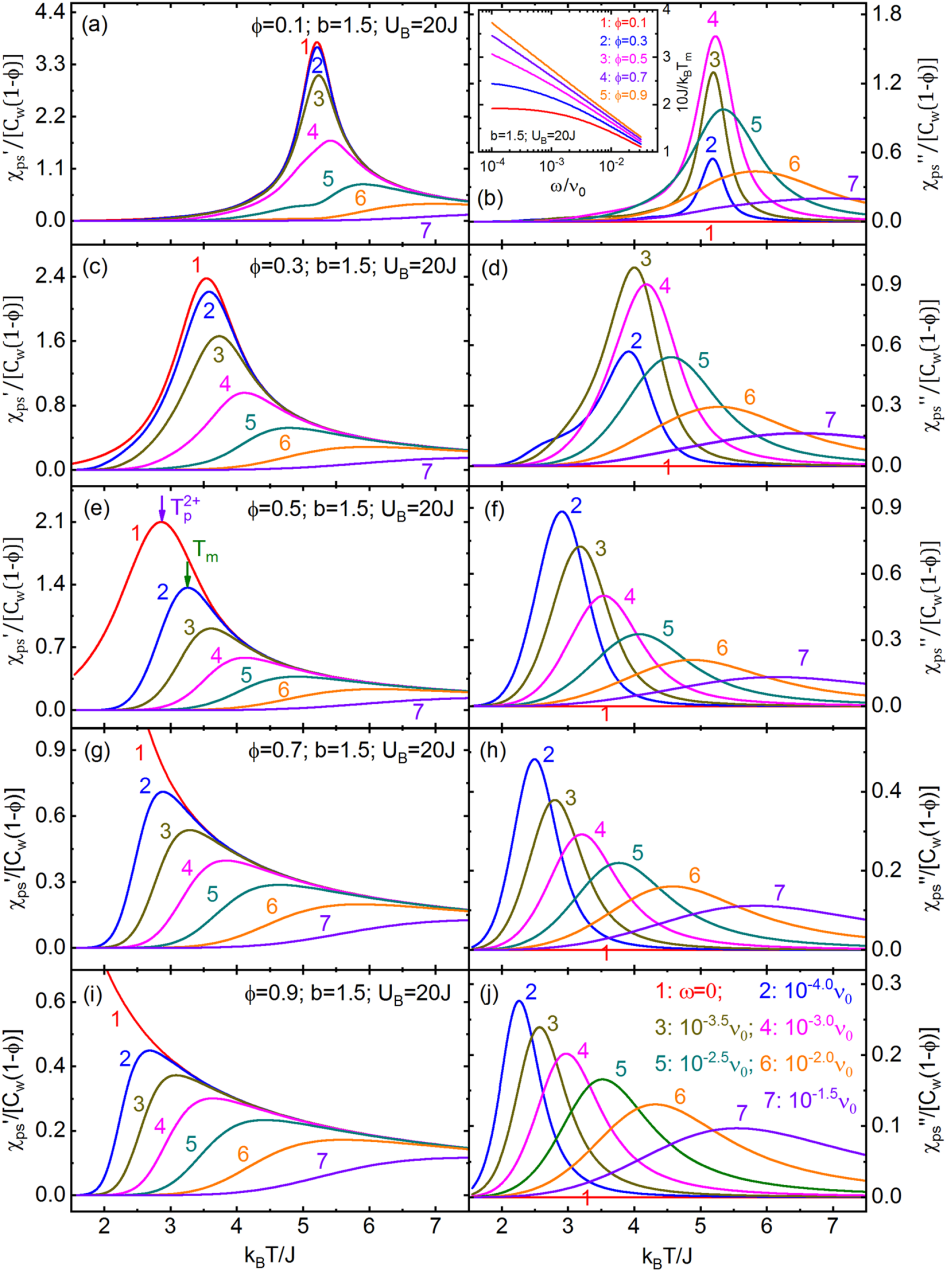
(**a**,**c**,**e**,**g**,**i**) ${{\chi }}_{ps}^{{\prime} }$ as well as (**b**,**d**,**f**,**h**,**j**) ${{\chi }}_{ps}^{{\prime\prime} }$ of 3D-ERSIGM with $b=1.5$, ${U}_{B}=20\,J$, and serial *ϕ* vs $T$ and serial ω. (**a**,**b**) *ϕ* = 0.1. (**c**,**d**) *ϕ* = 0.3. (**e**,**f**) $\phi =0.5$. (**g**,**h**) *ϕ* $=0.7$. (**i**,**j**) *ϕ* $=\,0.9$. ω $=\,0$ (red solid line), ${10}^{-4.0}{\nu }_{0}$ (blue), ${10}^{-3.5}{\nu }_{0}$ (deep yellow), ${10}^{-3.0}{{\rm{\nu }}}_{0}$ (pink), ${10}^{-2.5}{\nu }_{0}$ (dark green), ${10}^{-2.0}{{\rm{\nu }}}_{0}$ (orange), and ${10}^{-1.5}{{\rm{\nu }}}_{0}$ (violet). *T*_*m*_ in (**e**) is the temperature corresponding to the maximum of ${{\chi }}_{ps}^{{\prime} }$. The inset of (**b**) shows the corresponding $1/{T}_{m}$ vs ω.

Figure 7 shows the calculated ${{\chi }}_{ps}^{{\prime} }$ and ${{\chi }}_{ps}^{\,{\prime\prime} }$ of 3D-ERSIGM for $b=1.5$, ${U}_{B}=20\,J$, and $\phi =0.1,$ 0.3, 0.5, 0.7, 0.9 vs $T$ and ω by Eq. 12. The line 5 of Fig. 7a has two peaks of ${{\chi }}_{ps}^{{\prime} }$ for $\phi =0.1$, which is a typical characteristic of the critical-relaxation^56,57^. Moreover, compared with other *ϕ* values, the peak temperature (*T*_*m*_) of ${{\chi }}_{ps}^{{\prime} }$ for $\phi =0.1$ changes a little when ω is small, i.e. *T*_*m*_ vs ω follows the power-law $(\frac{1}{{T}_{m}-{T}_{p}^{2+}}\sim \omega )$ approximately as shown by the line 1 in the inset of Fig. 7b, which is another characteristic of the critical-relaxation^56,57^. These results indicate furtherly the definition rationality of ${\phi }_{e}=0.12$ (Fig. 4g,h) as the characteristic concentration of the evolution between the normal-ferroelectrics and RFEs.

As indicated obviously in the inset of Fig. 7b, *T*_*m*_ vs ω for $\phi =0.7,\,0.9$ agrees well with the Arrhenius-relation, and that for $\phi =0.3,\,0.5$ is between the power-law and Arrhenius-relation, which can be described by the Vogel-Fulcher-law $[\frac{1}{{T}_{m}-{T}_{v}} \sim \,\mathrm{ln}({\omega })]$ approximately, a typical characteristic of RFEs^31–33^, which originates from the effective interface-effect of PS clusters (App. B of SI) and the broad interaction distribution between the clusters (Fig. 2d). These results also confirm the rationality that ${\phi }_{p}$ is taken as the characteristic concentration of the evolution between the RFEs and paraelectrics (Fig. 4g,h). Based on Eq. 12 and Fig. 2, it could be expected that the distribution of relaxation time of ${{\chi }}_{ps}^{\,\ast }$ will first broaden and then narrow with increasing $\phi$, as shown in Fig. 7.

### Burns-transformation

Currently, the interpretation to Burns-transformation of high-temperature thermal-strain (${s}_{kl}^{T}$, $k,l=1,2,3$) and refractive-index (*n*_*kl*_) in RFEs is based on the macroscopic quadratic-electro-strictive and Kerr (quadratic-electro-optic) effects^14,42^, and Burns *et al*.^42^ proposed that the transformation stems from the appearance of PNRs at *T*_*b*_ being much higher than the DPT^15,16^. However, an unsolved problem is that the calculated spontaneous-polarizations by the above effects are much larger than the data of hysteresis and pyroelectric measurements^14,42^.

Theoretically, the appearance of *P*_*s*_ will inevitably lead to the deviations of ${s}_{kl}^{T}$ and *n*_*kl*_ from the high-temperature values. However, this does not rule out the possibility that other factors may also contribute to the transformation. In this paper, according to the coupling between PSs and crystal lattices in 3D-ERSIM, we give a new micro-mechanism of Burns-transformation, which stems from not only the PNRs but also ${\zeta }_{ni}^{ge}$ (App. F of SI) between PSs.

In view of the local-distortion (LD) of crystal lattices and the change of local-electronic-clouds (LE) induced by the local-interaction (LI) between the nearest-neighbor PS pairs in 3D-ERSIM, abbreviated as LI-LD and LI-LE couplings, respectively, and under the linear coupling approximation, the high-temperature ${s}_{kl}^{T}$ and *n*_*kl*_ of RFEs are (App. J and K of SI),

13a $${s}_{kl}^{T}-{s}_{kl}^{0}({T}_{r})\approx {\alpha }_{kl}(T-{T}_{r})-{c}_{kl}\frac{{u}_{ps}}{J}$$

13b $${n}_{kl}-{n}_{kl}^{0}({T}_{r})\approx {b}_{kl}(T-{T}_{r})-{d}_{kl}\frac{{u}_{ps}}{J}$$

Among them, *c*_*kl*_ and *d*_*kl*_ are the LI-LD and LI-LE coupling constants; ${s}_{kl}^{0}$,$\,{n}_{kl}^{0}$, *α*_*kl*_ and *b*_*kl*_ are the thermal-strain, refractive-index, the high-temperature thermal-expansion and thermo-optic coefficients independent of the LI-LD and LI-LE couplings^14,42^, respectively; and *T*_*r*_ is a reference temperature.

When the temperature is high enough, ${u}_{ps}\to 0$ (Eq. 5d and Fig. 4c), so ${s}_{kl}^{T}-{s}_{kl}^{0}({T}_{r})\approx {\alpha }_{kl}(T-{T}_{r})$ and ${n}_{kl}-{n}_{kl}^{0}({T}_{r})\approx {b}_{kl}(T-{T}_{r})$. With decreasing *T*, *u*_*ps*_ decreases, resulting in the deviation of ${s}_{kl}^{T}$ and *n*_*kl*_ from their linear behaviors of high-temperatures as shown in Fig. K1 of App. K in SI. Therefore, according to our theory (Eq. 13a,b), Burns-transformation originates from both the PNRs and ${\zeta }_{ni}^{ge}$ between PSs for *u*_*ps*_ is related to not only ${\eta }_{ne}^{g}$ but also ${\zeta }_{ni}^{ge}$ (Eq. 4c). In addition, the *T*_*b*_ increases as *c*_*kl*_ and $|{d}_{kl}|$ go up (Fig. K1 of App. J-K in SI).

### Comparisons with experimental results

**Figure 8:**
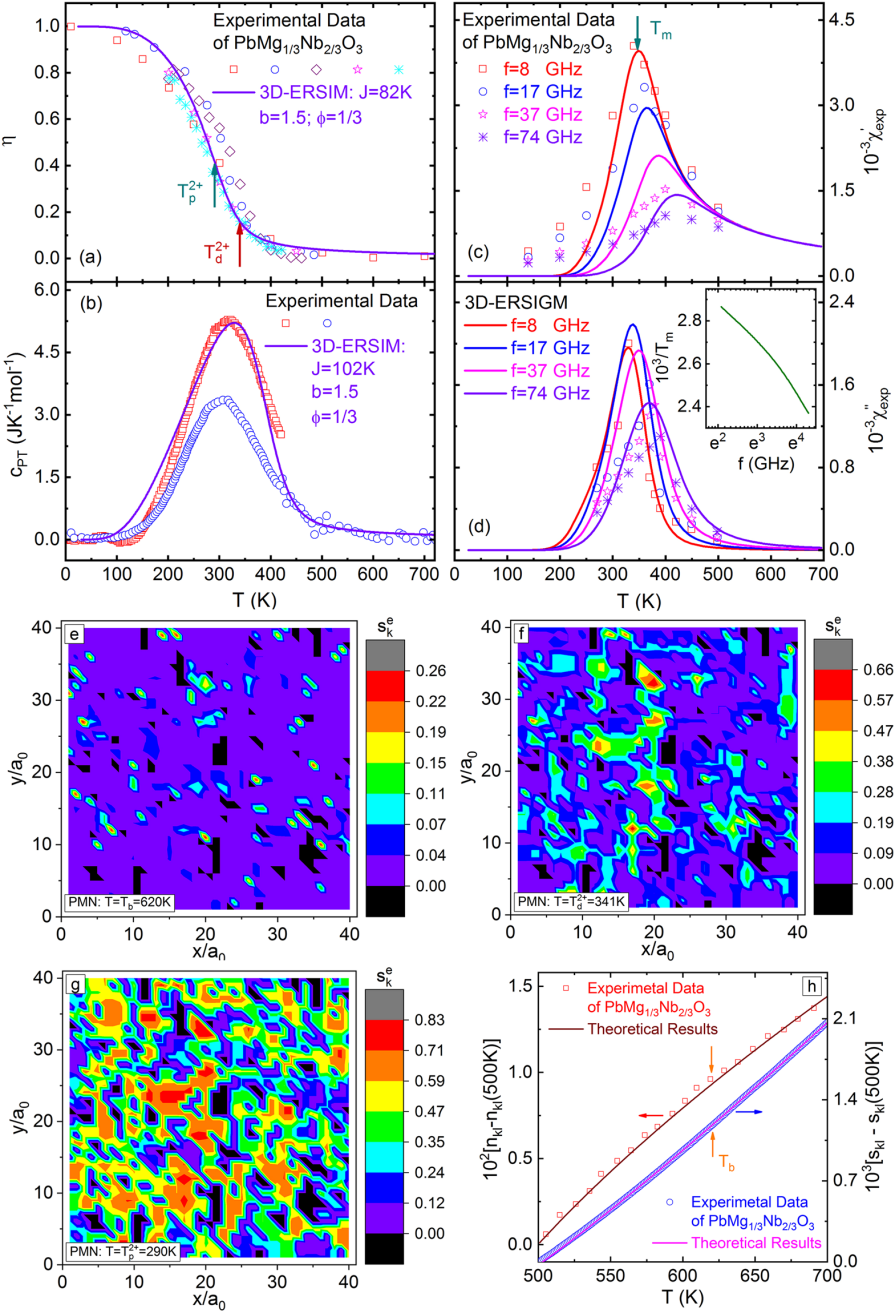
(**a**) Red square, blue circle [from Gehring *et al*. *Phys*. *Rev*. *B* **79**, 224109 (2009)], violet diamond, pink star, and cyan asterisk [from Stock *et al*. *Phys*. *Rev*. *B* **81**, 144127 (2010)] points are the experimental data of the order-parameter (*η*) of PMN single crystals vs temperature (*T*). The line is the results of 3D-ERSIM with *ϕ* = 1/3, $J=82\,{\rm{K}}$, and $b=1.5$. ${T}_{p}^{2+}=290\,{\rm{K}}$ and ${T}_{d}^{2+}=341\,{\rm{K}}$. (**b**) Red square [from Moriya *et al*. *Phys*. *Rev*. *Lett*. **90**, 205901 (2003)] and blue circle [from Tachibana *et al*. *Phys*. *Rev*. *B* **80**, 094115 (2009)] points are the experimental data of the specific-heat (*c*_*pt*_) of the DPT of PMN single crystals vs *T*. The line is the results of 3D-ERSIM with *ϕ* = 1/3, $J=102\,{\rm{K}}$, and$\,b=1.5$. (**c**) Points are the experimental data of the real part (${{\chi }}_{exp}^{{\prime} }$) and (**d**) imaginary part (${{\chi }}_{exp}^{{\prime\prime} }$) of complex-permittivity of PMN single crystals vs *T* for frequency (*f*) = 8, 17, 37, and 74GHz from Bovtun *et al*. [*J*. *Euro*. *Cer*. *Soc*. **26**, 2867 (2006)]. The lines are the results of 3D-ERSIGM with *ϕ *= 1/3, $J=87\,{\rm{K}}$, $b=1.5$, ${C}_{w}=3.28\times {10}^{3}\,{\rm{K}}$, ${U}_{B}=20\,J$, and ${\nu }_{0}=2.51\times {10}^{14}\,{\rm{H}}{\rm{z}}$. The inset of (**d**) shows the theoretical 1/*T*_*m*_ vs *f*. (**e**–**g**) Surface plots of the calculated ${s}_{k}^{e}$ in a x-y-plane of PMN when $T=620\,K\approx {T}_{b}$, $341\,K={T}_{d}^{2+}$, and $290\,K={T}_{p}^{2+}$ according to the 3D-ERSIM for *ϕ* = 1/3, $J=82\,{\rm{K}}$ and $b=1.5$. (**h**) Points are the experimental data of refractive-index (${n}_{kl}$, 4880Å) [red squares from H. S. Luo (private communication)] and thermal-strain (${s}_{kl}$) [blue circles from L. N. Wang (private communication)] of PMN single crystals vs *T*. The lines are our theoretical results (Eq. 13a-b), (i) for *n*_*kl*_: *ϕ* = 1/3, $J=90\,{\rm{K}}$, $b=1.5$, ${b}_{kl}=3.75\times {10}^{-5}$K^−1^, and$\,{d}_{kl}=0.124$, and (ii) for ${s}_{kl}$: *ϕ *= 1/3, $J=90{\rm{K}}$, $b=1.5$, ${\alpha }_{kl}=1.32\times {10}^{-5}$K^−1^, and ${c}_{kl}=9.81\times {10}^{-3}$.

At present, the commonly used methods for measuring *P*_*s*_ are hysteresis and pyroelectric measurements^14,34–36^. However, for the canonical RFE, PMN, the large external electric field used to polarize the samples will induce the structural phase-transition near 210K^34,36^, which leads to the measured data being not intrinsic. Here, we use the diffuse neutron scattering data (proportional to *η*) of PMN single crystals^37,38^ to compare with the results (Eq. 5a) of 3D-ERSIM, as shown in Fig. 8a. This plot indicates that the model with $\phi =1/3$, $J=82\,{\rm{K}}$, and $b=1.5$ gives a quite good fit to the data (${T}_{p}^{2+}=290\,{\rm{K}}$ and ${T}_{d}^{2+}=341\,{\rm{K}}$). Moreover, the model results show that PNRs appear well above ${T}_{p}^{2+}$ in PMN (Fig. 8e), and the predicted quasi-fractal characteristic of local-spontaneous-polarization (${s}_{k}^{e}$) of PMN near and below ${T}_{p}^{2+}$ (Fig. 8f,g) is also consistent with the experimental observations^17,18^.

The experimental data of the specific-heat of PMN, PbMg_1/3_Ta_2/3_O_3_, PbZn_1/3_Nb_2/3_O_3_, and Sr_x_Ba_1−x_NbO_6_ show that a specific-heat peak appears in RFEs^39–41^, which is also consistent with the model results as shown in Fig. 4c. In Fig. 8b, we give the comparison between the experimental data (*c*_*pt*_) of the specific-heat of PMN single crystals^39,41^ and our theoretical prediction of 3D-ERSIM with $\phi =1/3$, $J=102\,{\rm{K}}$, and $b=1.5$ (Eq. 5e). The fit looks reasonable, however the obtained values of *J* from *η* and *c*_*pt*_ have a relative deviation of ~20%. One possibly direct origination of this deviation is that the preceding *c*_*pt*_ data have big measurement error because the data were acquired by subtracting a huge nonlinear background vs *T* (~100 JK^−1^mol^−1^ at 300 K, and ~25 times larger than the *c*_*pt*_ peak height), and this background has some uncertainties, e.g. it was chosen differently by different researchers^39,41^, especially its downturn region with decreasing *T* overlaps with the *c*_*pt*_ peak (Fig. 6 of Tachibana *et al*.^41^), which leads to the obtained *c*_*pt*_ peak height and temperature of Moriya *et al*. being ~40% larger and ~7% higher than those of Tachibana *et al*.^41^, respectively. So, the background selection in PMN is a problem worthy of deep studies. Moreover, we would like to point out that this paper chooses to fit the whole *c*_*pt*_ peak of PMN^39^, resulting in the theoretical peak temperature of ~4% higher than that of the experiment, i.e. the corresponding $J$ value may be overestimated by ~4% (Fig. 8b). Another possible origination is the neglection of the mutual Coulomb interaction between PNRs^72^ in our theory. E.g., this interaction will induce the adjacent PNRs to tend to the reverse arrangement of spontaneous polarization, leading to the decrease or even disappearance of the local-order-parameter (${s}_{k}^{e}$) in their adjacent parts (similar to the domain walls in normal-ferroelectrics), i.e. the reduction of $\eta$. Moreover, it is worth pointing out that, in addition to ${s}_{k}^{e}$, *c*_*pt*_ is related to the correlation-function (${\zeta }_{nk}^{ge}$) (Eq. 4c,d). It could be imagined that the above interaction would also result in the decrease of ${\zeta }_{nk}^{ge}$. As for the quantitative reduction of ${s}_{k}^{e}$ and ${\zeta }_{nk}^{ge}$, further researches are needed. Of course, other possible mechanisms may induce the deviation, too.

Due to the spatial distribution of ${s}_{k}^{e}$ (Fig. 5), the phase boundaries, sub-phase boundaries, and domain walls^68–70^ appear during the DPT of RFEs, and these movable boundaries and walls will contribute significantly to the complex-permittivity (collectively referred as ${{\chi }}_{b}^{\,\ast }$) below the transition temperature (${T}_{p}^{2+}$)^15,73–75^. Therefore, the main contributions of the complex-permittivity of RFEs are, respectively, ${{\chi }}_{b}^{\,\ast }$ when $T < {T}_{p}^{2+}$ or ${{\chi }}_{ps}^{\,\ast }$ when $T > {T}_{p}^{2+}$. For PMN, ${T}_{p}^{2+}=290\,{\rm{K}}$ (Fig. 8a), and according to the experimental data of complex-permittivity (${{\chi }}_{exp}^{\,\ast }={{\chi }}_{exp}^{{\prime} }-{i}_{c}{{\chi }}_{exp}^{\,{\prime\prime} }$), the frequency (*f*) is about 1GHz when the peak temperature (*T*_*m*_) of ${{\chi }}_{exp}^{{\prime} }$ is equal to ${T}_{p}^{2+}$ (Bovtun *et al*.^32^). Therefore, the ${{\chi }}_{exp}^{\,\ast }$ of PMN is mainly ${{\chi }}_{ps}^{\,\ast }$ for *f* > 1GHz. In Fig. 8c,d, we give the comparison of ${{\chi }}_{exp}^{\,\ast }$ of PMN single crystals between 8 and 74GHz^32^ to the results (Eq. 12) of 3D-ERSIGM with $\phi =1/3$, $J=87\,{\rm{K}}$, $b=1.5$, ${C}_{w}=3.28\times {10}^{3}\,{\rm{K}}$, ${U}_{B}=20\,J$, and ${\nu }_{0}=2.51\times {10}^{14}\,{\rm{H}}{\rm{z}}$. The theoretical $1/{T}_{m}$ vs *f* is shown in the inset of Fig. 8d, and it varies as the Vogel-Fulcher-law. In view of that ${{\chi }}_{exp}^{\,\ast }$ has large measurement errors when *f* > 1GHz, especially ${{\chi }}_{exp}^{\,{\prime\prime} }$^32,76^, the theoretical and experimental results can be considered being consistent with each other.

Figure 8h shows the fits of the theoretical predictions (Eq. 13a,b) to experimental data near *T*_*b*_, (i) for *n*_*kl*_: $\phi =1/3$, $J=90\,{\rm{K}}$, $b=1.5$, ${b}_{kl}=3.75\times {10}^{-5}\,{{\rm{K}}}^{-1}$, and$\,{d}_{kl}=0.124$, and (ii) for *s*_*kl*_: $\phi =1/3$, $J=90\,{\rm{K}}$, $b=1.5$, ${\alpha }_{kl}=1.32\times {10}^{-5}\,{{\rm{K}}}^{-1}$, and ${c}_{kl}=9.81\times {10}^{-3}$. The good fits illustrate that our theory not only provides a new quantitative micro-mechanism of Burns-transformation, but also avoids the too large spontaneous-polarization data obtained by the quadratic-electro-strictive and Kerr effects (Eqs. J3 and K3 of Apps. J and K in SI that do not consider the contribution of ${\zeta }_{ni}^{ge}$ as shown in Eq. 13a,b) when compared with the hysteresis and pyroelectric measurements^14,42^.

The experimental results show that there is an intermediate temperature (T*) between ${T}_{p}^{2+}$ and *T*_*b*_ in RFEs, and according to the current view, T* is the temperature where PNRs change from the high-temperature dynamic to low-temperature static^38,76^. Based on our theory, the relaxation or fluctuation time of the PNRs near *T*_*b*_ is much smaller than that near ${T}_{d}^{2+}$ (Insets of Figs. 7b and 8d), and ${T}_{d}^{2+}$ is the characteristic temperature that the PNRs are nearly individual above it (Figs. 5e,f and 8e), while they interconnect to form the quasi-fractal structure of local-spontaneous-polarization below it (Figs. 5d–b and 8f,g), so T* just corresponds to ${T}_{d}^{2+}$ as shown in Fig. 8a, and the only difference is their mathematic definitions.

Since x can be continuously adjusted from 0 to 1^49,51^, BZ_x_T_1−x_ is an ideal system to verify our theory. However, due to the limitation of crystal growth technology, large-size and high-quality single crystals with x > 0.2 cannot be grown so far^54^, which has affected the measurements of some physical parameters (especially high-frequency permittivity >GHz) to some extent. Nevertheless, with considering the similarity of the low- to high-frequency permittivity of PMN, the analogy of the phase diagram of BZ_x_T_1−x_ given by the low frequency (100Hz-500kHz) permittivity^49,51^ with our theoretical results has a certain degree of rationality. The obtained phase diagram shows that, with increasing x, BZ_x_T_1−x_ evolves from the normal-ferroelectrics to RFEs to paraelectrics (Fig. 11 of Maiti *et al*.^49^), while the *T*_*m*_ vs ω from the power-law (Fig. 1 of Kleemann *et al*.^51^) to Vogel-Fulcher-law to Arrhenius-relation (Fig. 8 of Maiti *et al*.^49^), which is consistent with our theoretical predictions as shown in Fig. 4g and the inset of Fig. 7b.

It is worth pointing out that the results of the existing Monte-Carlo-simulations of 2D and 3D-RSIM^25–28^ support our PSS-MF method as shown in the SI 6.

### Definition of canonical RFEs

PMN is generally viewed as the canonical RFE, but what is a canonical RFE is not well defined so far^52,53^. The experimental results show that the systems with definite components but adjustable ion distributions, such as PbSc_1/2_Ta_1/2_O_3_, continuously evolve from normal-ferroelectrics to RFEs with the increase of disorder^14^, so it looks reasonable that the random distribution of ions, i.e. the most disordered case, is one necessary condition of canonical RFEs. Clearly, the 3D-ERSIM (Eq. 1a) proposed in this paper satisfies this condition.

Phenomenologically, the order-parameter (Fig. 4a), local-order-parameter (Fig. 5b-f), specific-heat (Fig. 4c), complex-permittivity (Fig. 7c-d), and average relaxation time (Inset of Fig. 7b) of 3D-ERSIGM near *ϕ*_*c*_ have the general characteristics of relaxor-ferroelectricity^17,18,31–41^. Moreover, when *ϕ* = *ϕ*_*c*_, ${g}_{p}^{n}/n$ is nearly independent of *n* (Fig. 2d), which means that, compared with other *ϕ*, the average transition temperatures (Eq. 4 and Fig. 2d) of the PS clusters in 3D-ERSIM of *ϕ*_*c*_ are the most uniform except for the case near *ϕ* = 0. Furthermore, *ϕ*_*c*_ is the percolation threshold of PS-vacancies in 3D-RSIM, one of the most characteristic values of this model^67^. Therefore, here we propose to define that an RFE can being described by 3D-ERSIGM of *ϕ* = *ϕ*_*c*_ is the canonical (Fig. 4g-h), and based on this definition, PMN (*ϕ* = 1/3 according to Fig. 8) is the RFE quite close to the canonical.

## Discussion

By the fitting of our theoretical results to the experimental data (Fig. 8), the obtained *ϕ *= 1/3 is a reasonable value for PMN. In addition, we can conclude that PbZn_1/3_Nb_2/3_O_3_ and PbMg_1/3_Ta_2/3_O_3_ also correspond to 3D-ERSIGM of *ϕ* = 1/3. Moreover, it could be imagined that, for BZ_x_T_1−x_, *J*, *μ*, and *U*_*B*_ are almost irrelevant to x when x is small. However, due to the RISF and when x is large enough, *J*, *μ*, and *U*_*B*_ will change with increasing x, which needs deep studies. For the more complicated and anisotropic RFE, Sr_x_Ba_1−x_Nb_2_O_6_, the relationship of *ϕ*, *J*, *μ*, and *U*_*B*_ with x requires future researches, too.

It could be expected that the domain structure, including domain walls, can be obtained based on the calculated ${s}_{k}^{e}$ (Figs. 5b–f and 8e-g) and the theory of domain formation^68–70^. Moreover, the method for calculating ${{\chi }}_{ps}^{\,\ast }$ of single-PS flipping in this paper (Sec. Complex-permittivity…) would lay the foundation to obtain ${{\chi }}_{b}^{\,\ast }$ of the overall movement of PSs, i.e. the multi-PS flipping, in the phase boundaries and domain walls^73–75^. Of course, they are the issues that need further studies. We would like to point out that the mutual Coulomb interaction of PNRs at *T* < T* has recently been evidenced by Kleemann and Dec^72^, to give rise to a super-dipolar glass state below the glass transition temperature (=240 K) in PMN, and the relevant research of ${{\chi }}_{b}^{\,\ast }$ will undoubtedly deepen the understanding of this state.

It would be speculated that the approximation to calculate *P*_*s*_ (Eq. 5b), ${{\chi }}_{s}^{\,ps}$ (Eq. 5c), and ${{\chi }}_{ps}^{\,\ast }$ (Eq. 12) has large errors when *ϕ* is high, so it is a potential work to explore more accurate calculation method. Other future studies are: (i) To generalize the PSS-MF for solving isotropic 3D-ERSIGM to anisotropic cases, so that the possible anisotropic RFEs, such as the single-axis tungsten bronze and layered Aurivillius structures^17^, can be described; (ii) Coupling of PSs with crystal lattices and the acoustic properties^16^ as well as possible structural phase-transitions of RFEs^34,36^; (iii) To model the ultrahigh piezoelectric RFE, PMN-PbTiO_3_^52,53,77–79^, a heterogeneous system of *J* and *μ*; and (iv) To generalize our theory to the corresponding ferromagnetic systems, in particular spin-glasses, etc.^20–22^.

As shown in Figs. 1a, 4, 5, 7 and 8, our theory predicts that RFEs are a special kind of ferroelectrics (observable macro-spontaneous-polarization) with DPT originating from the spatial-dynamical heterogeneity as indicated by the spatial variation of local-spontaneous-polarization [local-order-parameter (${s}_{k}^{e}$)] including the coexistence of para- and ferroelectric regions (Fig. 5c) and corresponding distribution of relaxation time (Figs. 6a,b and 7c–f), which leads to ${s}_{k}^{e}$ being the key parameter of relaxor-ferroelectricity, instead of the macro-spontaneous-polarization [order-parameter (*η*)]. In this respect, normal-ferroelectrics are only special ferroelectrics where ${s}_{k}^{e}$ is equal everywhere. It is worth noting that at low temperature (*T* → 0), there are macro-spontaneous-polarization regions throughout RFEs (Fig. 5b) according to the percolation theory^67^, i.e. long-range ferroelectric order exists in RFEs although it is different from the spatial uniform one in normal-ferroelectrics. In addition, there are domain walls^17,18^ in RFEs as described in Sec. Local-order-parameter, and by analogy with the experimental results in normal-ferroelectrics^73,74^, the relaxation time of their lateral movement should increase with decreasing temperature and tends to infinity when *T* → 0, resulting in the freezing or glass transition of domain wall movement at a certain temperature^3,72–74^. In short, based on the present theory, RFEs have spatially-dynamically heterogeneous local-order-parameter and the corresponding mesoscopic defects (domain walls and phase boundaries etc.) of the local-order-parameter show glassy behavior. Of course, the correctness of this relaxor-ferroelectricity picture still needs further theoretical and experimental studies.

The authors would like to point out that, in the preceding theories or models (SI 1), the spherical random-field random-bond model of Pirc *et al*.^7,8^ and the soft-mode theory with random-electric-field of Arce-Gamboa and Guzmán-Verrí^10^ give the most quantitative predictions. The theory of Arce-Gamboa and Guzmán-Verrí gives that: (1) For weak random-electric-field, long-range ferroelectric order sets in at a transition temperature where the obtained *η* changes discontinuously and it is accompanied by metastable paraelectric or random-electric-field state down to *T* → 0; (2) For moderate one, there is no transition as the paraelectric state becomes stable at all temperatures and the long-ranged polar state is now metastable; and (3) For strong one, only the paraelectric state exists. According this theory, PMN belongs to the case of (1)^10^, so both the theoretically discontinuous change of *η* with *T* in the absence of an external electric field and no PNR above the transition are inconsistent with the experimental results^18,37,38^, which is obviously different to the predictions of the present theory (Fig. 8a,e). The physical origination of these differences is that, according to our theory, there are dipole- or PS-vacancies randomly distributing in RFEs (Eq. 1a and Fig. 1a) so that the interfaces between PS and PS-vacancy groups appear, which leads to the dispersion of the phase transition^64^, i.e. interface-effect (App. B), but similar interface-effect term does not exist in the model Hamiltonian as shown in Eq. 1 of Arce-Gamboa and Guzmán-Verrí theory^10^.

The spherical random-field random-bond model predicts that the DPT corresponds a dipolar glass transition, however, in view of the elementary motion and interaction unit being the effective dipole, i.e. PNR, this model is at the mesoscopic or semi-microscopic level and the glass transition might correspond to the super-dipolar glass transition of PNRs^72^, which is an issue of the present theory to be further explored as mentioned in the 2^nd^ paragraph of this section.

## Supplementary information

Supplementary Information.
