# Supplementary material for: Theory of relaxor-ferroelectricity
Source: Sci Rep. 2020 Mar 19;10:5060. doi: 10.1038/s41598-020-61911-5 (PMC7081360; doi:10.1038/s41598-020-61911-5)
Supplement: Supplementary file 1 — Supplementary Information. [file 41598_2020_61911_MOESM1_ESM.pdf]

Supplementary Information

Theory of Relaxor-Ferroelectricity

Li-Li Zhang[1,2] and Yi-Neng Huang[1,2]

[1] National Laboratory of Solid State Microstructures, School of Physics, Nanjing University, China. [2] Xinjiang Laboratory of Phase Transitions and Microstructures in Condensed Matters, College of Physical Science and Technology, Yili Normal University, China.

Supplementary Information 1

Although no existing theory can quantitatively describe the main facts of relaxor-ferroelectricity till now, there are some influencing ones:

1. *Component disorder model*. Smolenskii calculated the distribution of micro-components, proposing this model to obtain the static permittivity of RFEs based on the assumption that the local phase-transition temperature is proportional to the micro-concentration[2,14]. To make this model better, the assumption needs to be studied further on a microscopic level.
2. *Random-interaction random-field theory*. This theory is proposed by Kleemann et al.[3,4], and usually called as the random-field theory. It emphasizes the influence of such field to the phase-transition and domain structures, but is lack of quantitative predictions to compare with experimental results.
3. *Polar glass model*. Based on the coupling between polar-nano-regions (PNRs) first proposed by Burns et al.[14-16,42], Viehland et al.[5,6] suggested this model. Although the model is very inspiring for exploring the microscopic mechanism of relaxor-ferroelectricity, they did not give a clear model Hamiltonian.
4. *Spherical random-field random-bond model*. Pirc et al.[7,8] proposed this model and treated the PNRs as effective dipoles, assuming they are in a random Gaussian internal field with their interaction also following a Gaussian distribution. This model successfully predicts the ferroelectric phase-transition and effective dipole glass-transition with specific parameters. However, it has an unphysical assumption that the effective dipoles have an infinite long and strong interaction. To make this theory better, we can further calculate the dynamics, especially the complex permittivity of the system, as well as writing down the distributions of the PNRs, the random-field, and random-bond on a microscopic level.
5. *Soft pseudospin glass theory*. This theory is presented by Sherrington[9], but still lack of quantitative predictions so far.
6. *Soft-mode theory with random-electric-field*. Arce-Gamboa and Guzmán-Verrí[10] established this theory by introducing a random-electric-field into the soft-mode theory of the normal-ferroelectric phase-transition. The physical image of this theory is clear, but the predicted order-parameter changes discontinuously with temperature in the absence of an external electric field, which is inconsistent with the experimental results, and the relevant origination still unclear now.

Supplementary Information 2

RFEs are systems in which two or more kind ions are randomly distributed on crystal lattices[14-19], e.g. Mg^{2+} and Nb^{5+} in PMN, Sc^{3+} and Ta^{5+} in $PbSc_{1/2}Ta_{1/2}O_3$, and Zr^{4+} and Ti^{4+} in BZ_xT_{1-x}. According to the disorder ion valence, it is divided into isovalent (such as BZ_xT_{1-x}) and heterovalent (PMN and $Sr_xBa_{1-x}Nb_2O_6$) RFEs. Due to the differences in the size and charge of the disorder ions, both the random-internal-stress-field (RISF) and random-internal-electric-field (RIEF) are generated in the heterovalent RFEs, but there is only RISF in the isovalent ones[4]. It is worth to point out that the RIEF defined by Kleemann et al.[4] is different to the random-electric-field by Arce-Gamboa et al.[10]. In fact, according to the Hamiltonian (Eq.1 of Arce-Gamboa et al.[10]), the random-electric-field contains the contributions of all randomness in RFEs, including the RISF, RIEF, and part of the random interaction between PSs (Eq.1 of Kleemann et al.[4]).

According to the fact that BZ_xT_{1-x} and $Sr_xBa_{1-x}Nb_2O_6$ evolve from normal-ferroelectrics to RFEs with increasing x[48-51], it is feasible to obtain a reasonable theory of relaxor-ferroelectricity by appropriately introducing component disorder into the preceding theories of normal-ferroelectricity. There are two successful schemes to describe normal-ferroelectricity, including the PS[11,12] and soft-mode[13]. Here, we choose the first one because it is convenient and direct to introduce the component disorder. The PS scheme includes the 3D-Ising-model and its extended ones[11,12]. The following takes BZ_xT_{1-x} and PMN as examples to construct the extended 3D-Ising-model with disordered components.

Model Hamiltonian of BZ_xT_{1-x}. For the convenience of descriptions, in BZ_xT_{1-x}: (i) The permanent electric dipole-moments of the unit cells containing Zr^{4+} and Ti^{4+} are expressed as μ_Z and μ_T, and the corresponding PSs as σ_Z and σ_T; and (ii) The interaction energy constants between the nearest-neighbor PS pairs of $\sigma_Z\sigma_Z$, $\sigma_T\sigma_T$ and $\sigma_Z\sigma_T$ as J_{ZZ}, J_{TT}, and J_{ZT}, respectively. According to the experimental results: (a) The paraelectric-ferroelectric phase-transition temperature (T_{c1}) of $BaTiO_3$ (BT) is approximately 380K, and the Curie-Weiss constant of the paraelectric phase (C_w) $\approx 1.6\times10^5$K[54]; and (b) There is not any phase-transition of $BaZrO_3$ (BZ) from 2K to 1375K, and the Curie constant of BZ (C_c) $\approx 3.2\times10^3$K[55]. According to the Weiss, i.e. single-PS, mean-field of 3D-Ising-model[58,59], it is obtained that: (i) $J_{TT}\approx T_{c1}/6$ =63K, and $J_{ZZ}\approx$0K; and (ii) $\mu_Z/\mu_T=\sqrt{C_c/C_W}=0.14$, so we can assume $\mu_Z\approx 0$, $\sigma_Z\approx 0$, and $J_{ZT}\approx 0$ accordingly. $\sigma_Z=0$ is equivalent to the absence of PSs, i.e. PS-vacancies, in the unit cells containing Zr^{4+}. So, before considering the RISF, BZ_xT_{1-x} can be simplified as the following 3D-random-site-Ising-model (3D-RSIM):

1. The crystal lattice of the model is simple cubic. Some lattice points are randomly occupied by the permanent electric dipoles (permanent dipole-moment being $\mu=\mu_T$) of the Ti^{4+} cells with the concentration being $1-\phi$, and the rest are the dipole-vacancies of the concentration ϕ ($\phi=x$).
2. The orientational motion of the permanent dipoles are equivalent to the two states of PSs[11,12], σ_k is used here to represent the kth-PS in the model, and its two states are labeled by $\sigma_k=\pm1$. The dipole-vacancies are equivalent to having no PS, i.e. PS-vacancies.
3. Only the interaction between the nearest-neighbor PS pairs is not zero, and the interaction energy between the kth- and its nearest-neighbor lth-PS is $-J\sigma_k\sigma_l$, where $J\equiv J_{TT}$.

The corresponding model Hamiltonian can be described by the following 3D-RSIM[25-28],

$$H_{RSIM}=-J\sum_{k\neq l}^{\{nn\}}\sigma_k\sigma_l r_k^{\phi} r_l^{\phi} \tag{S2.1}$$

Moreover, according to Kleemann et al.[4], the Hamiltonian (H_{RISF}) related to the RISF can be written as the random Zeeman term,

$$H_{RISF}=-\sum_{k=1}^{N}S_k\sigma_k \tag{S2.2}$$

In which, S_k is the effective random Zeeman field related to the RISF, and $\langle S_k\rangle=0$, but $\langle {S_k}^2\rangle\neq0$, where $\langle\cdots\rangle$ means the average of the whole lattice points.

Therefore, the total model Hamiltonian of BZ_xT_{1-x} is,

$$H_{BZT}=H_{RSIM}+H_{RISF} \tag{S2.3}$$

Model Hamiltonian of PMN. In PMN: (i) The PSs of the unit cells containing Mg^{2+} and Nb^{5+} are expressed as σ_M and σ_N, and the corresponding electric dipole-moments as μ_M and μ_N, as well as $\phi=1/3$; and (ii) The interaction energy constants between the nearest-neighbor PS pairs of $\sigma_M\sigma_M$, $\sigma_N\sigma_N$ and $\sigma_M\sigma_N$ as J_{MM}, J_{NN}, and J_{MN}, respectively. Similar to BZ_xT_{1-x}, here we assume that $\mu_M\approx 0$, $\sigma_M\approx0$, $J_{MM}\approx0$, and $J_{MN}\approx0$, but $\mu_N\neq0$ and $J\equiv J_{NN}\neq0$. The Hamiltonian related to the random interaction between PSs has the same form as Eq.S2.1.

Moreover, besides the H_{RISF}, the model Hamiltonian (H_{RIEF}) related to the RIEF in PMN can also be written as another random Zeeman term[4],

$$H_{RIEF}=-\sum_{k=1}^{N}E_k\sigma_k \tag{S2.4}$$

where E_k is the effective random Zeeman field related to the RIEF, and $\langle E_k\rangle=0$, but $\langle {E_k}^2\rangle\neq0$.

Therefore, the model Hamiltonian of PMN is,

$$H_{PMN}=H_{RSIM}+H_{RISF}+H_{RIEF} \tag{S2.5}$$

That is Eq.1a in our paper.

As shown in Appendix B, the influences of S_k and E_k can be expressed as two factors of the effective interface-effect approximately, and the specific forms of S_k and E_k are not given here, which is a valuable question needing further studies for relaxor-ferroelectricity.

We would like to point out that the real ion distribution in BZ_xT_{1-x} and PMN may deviates from randomness more or less. Moreover, the detail modelling of other RFEs, such as $PbSc_{1/2}Ta_{1/2}O_3$ and $PbSc_{1/2}Nb_{1/2}O_3$ (adjustable distribution of ions on lattices)[14], PMN-$PbTiO_3$ (heterogeneous J and μ)[52,53,76-78], and $Sr_xBa_{1-x}Nb_2O_6$ (possible anisotropy of the interaction between PSs and the relation between ϕ and x)[17,31,33], need future works, too.

Supplementary Information 3

The solution of 3D-ERSIGM usually consists of calculating: (i) Thermodynamic parameters of 3D-ERSIM, such as the order parameter, local order parameter, static permittivity, and specific-heat; and (ii) Dynamic parameters, such as the complex permittivity, including the relaxation time and its distribution.

According to the author's knowledge, the exact partition function of 3D-RSIM (including 3D-Ising-model) has not yet been obtained. The approximate solving methods[60-62] are the renormalization-group-theory and finite-series-expansion. The starting point of the group-theory is that the system has the invariance under scale transformation, i.e. its correlation length must tend to infinity. However, whether this invariance exists in the 3D-RSIM with large ϕ still need further studies. The finite-series-expansion method for solving 3D-Ising-model is only accurate at either low- or high-temperature, and the calculation error is larger near the phase-transition. It would be expected that the randomness in 3D-RSIM will increase the error of this method further.

The problem of Monte-Carlo-simulations (MCSs)[25-28] to obtain the thermodynamic parameters of 3D-RSIM is similar to the molecular dynamic simulations[75], i.e. whether a sufficiently large simulation system close enough to the ideal equilibrium state is got after a number ($\sim 10^6$) of MCS steps, especially at low-temperature.

As for the complex permittivity of the RSIGM, the exact solutions of the 2- and 3-dimensional have not been obtained except for the 1-dimensional[29,30], and there is even no effective approximation method to solve it so far. In addition, the authors have not seen the corresponding results of MCSs.

In view of: (i) The successes of the existing multi-spin mean-field methods [such as Kramers-Wannier (finite-width-spin-strip)[63,66] and Bethe-Peierls (special-spin-cluster)[65,66]] for solving Ising-model, and especially, with increasing the spin number that the mean-fields contain, the corresponding results tend to the exact solution of 2D-Ising-model[64]; and (ii) Inspired by the exact solution of the complex permittivity of 1D-RSIGM[29,30], in order to solve 3D-ERSIGM, we propose here a new mean-field of PS-strings (PSSs) that contains more PSs and takes into account the correlation between PSs in 3D-space, and it is called the PSS-MF to distinguish from the mean-field of straight-spin-chains for solving 2D- and 3D-Ising-models[63,66].

Supplementary Information 4

Here, the maximum value (n_c) of n for numerical calculations is taken as where nq_n is 1% of its maximum and $n_c > n_p$ as shown in Table S4.1.

Table S4.1: n_p and n_c vs ϕ

ϕ	$\boldsymbol{n_p}$	$\boldsymbol{n_c}$
0.10	17	132
0.20	12	90
0.30	9	70
0.40	7	54
0.50	5	42
0.60	4	31
0.70	3	21
0.80	2	14
0.85	1	11
0.90	1	8
0.95	1	5
0.99	1	3

Supplementary Information 5

As shown in Fig.1c, the constructed PSSs are spatially anisotropic for each scan of the six kinds, which inevitably lead to the spatial anisotropy of s_{ni}^{ge}. In order to eliminate the anisotropy, the method to obtain s_k^e in this paper is: (i) First, calculating the corresponding $s_{ni}^{ge}(xyz)$, $s_{ni}^{ge}(yxz)$, $s_{ni}^{ge}(xzy)$, $s_{ni}^{ge}(zxy)$, $s_{ni}^{ge}(yzx)$, and $s_{ni}^{ge}(zyx)$ by the six scans; and (ii) Then, averaging them, that is,

$$s_k^e = \frac{1}{6}\left[s_{ni}^{ge}(xyz) + s_{ni}^{ge}(yxz) + s_{ni}^{ge}(xzy) + s_{ni}^{ge}(zxy) + s_{ni}^{ge}(yzx) + s_{ni}^{ge}(zyx)\right] \quad \text{(S5.1)}$$

Supplementary Information 6

In view of some controversial results of FPB-MDSs of RFEs[75], we have not presented their comparisons with our theory. To check the PSS-MF, we compare the data of order parameter, specific-heat, static permittivity obtained by the Monte Carlo simulations of 2D- and 3D-RSIM to our theoretical results.

Maybe due to the problem as pointed above (SI 3), there are only one set of MCS data of the order parameter and internal energy (with large fluctuations) of 3D-RSIM vs T, and ϕ is limited below 0.6 according to authors' knowledge[25]. However, the MCS data of the order parameter, static permittivity, and specific-heat of 2D-RSIM vs T are more systematic[26-28], and ϕ is from 0 to 0.7 ($\phi_p = 0.41$ for 2D-RSIM), which probably originates from the total simulation times (proportional to the MCS steps multiplying lattice points) of 2D-RSIM being much less than 3D-RSIM. Nevertheless, taking into account the qualitative consistency of the thermodynamic parameters of 2D and 3D-RSIM[25-28], below

we will compare the results of 3D-ERSIM obtained by our PSS-MF method with the MCSs of 2D-RSIM.

The MCS data of 2D-RSIM indicate that: (i) For ϕ is much smaller than ϕ_{p}, there is only one peak of specific-heat (Fig.2 of Selke et al.[26] and Fig.3 of Kutlu et al.[28]) and static permittivity (Fig.2 of Fulco et al.[27]), and the peak height and position decrease, while the peak broadens and the dispersity of order parameter becomes stronger (Fig.1 of Fulco et al.[27]) with increasing ϕ; (ii) For $\phi \sim \phi_{\mathrm{p}}$, two peaks of specific-heat (Fig.2 of Selke et al.[26]) and static permittivity (Fig.2 of Fulco et al.[27]) appear, while the dispersity of order parameter becomes more stronger (Fig.1 of Fulco et al.[27]) as ϕ goes up; and (iii) For ϕ is larger than ϕ_{p}, there is only one broad peak of specific-heat (Fig.2 of Selke et al.[26]) and the peak height decreases with the increase of ϕ. The above results are consistent with those of our PSS-MF of 3D-ERSIM (Fig.4).

We would like to point out that the phase-transition temperature of 2D-RSIM with $\phi = \phi_{\mathrm{p}}$ being zero is only a guess, i.e. there is no supporting MCS datum (Fig.4 of Fulco et al.[27] and Fig.5 of Kutlu et al.[28]). If we give up this speculative point, the phase diagram of 2D-RSIM as shown in Fig.1of Selke et al.[26] agrees with Fig.4g.

It should be noted that, for such a simple 2D-RSIM, there are also significant differences in the MCS results of the same ϕ. For example, Fig.3 of Kutlu et al.[28] shows that the specific-heat peak has been clearly diffused when $\phi = 0.05$, but the peak is still sharp enough even though $\phi = 0.15$ in Fig.2 of Selke et al.[26]. Nevertheless, the results of the MCSs of 2D-RSIM and the PSS-MF of 3D-ERSIM are mutually validated on the whole.

Appendix A: Glauber transition probability

Based on the detailed balance condition[29,30] of the Hamiltonian (Eq.1a), it is obtained that,

$$w(\sigma_k) = \nu \frac{\exp\left(-\frac{\sigma_k F_k}{k_B T}\right)}{\exp\left(-\frac{\sigma_k F_k}{k_B T}\right) + \exp\left(\frac{\sigma_k F_k}{k_B T}\right)} \tag{A1}$$

By $\exp\left(\pm\frac{\sigma_k F_k}{k_B T}\right) = \cosh\left(\frac{F_k}{k_B T}\right) \pm \sigma_k \sinh\left(\frac{F_k}{k_B T}\right)$, we get,

$$w(\sigma_k) = \frac{\nu}{2}\left[1 - \sigma_k \tanh\left(\frac{F_k}{k_B T}\right)\right] \tag{A2}$$

For the Hamiltonian of Eq.2b, the local field (F_{ni}^g) of σ_i^s in n-g-PSSs is,

$$F_{ni}^g \equiv J\left\{\sigma_{i-1}^s + \sigma_{i+1}^s + \frac{g}{n}\left[\left(1 - \frac{1}{n^b}\right)\eta_n^g + \frac{1}{n^b}\right]\right\} \tag{A3}$$

where $i = 1, \cdots n$, and $\sigma_0^s = \sigma_{n+1}^s = 0$. Therefore:

For $n = 1$,

$$w_1(\sigma_1^s) = \frac{\nu}{2}(1 - \gamma\sigma_1^s) \tag{A4}$$

For $n = 2$,

$$\begin{cases} w_2(\sigma_1^s) = \frac{\nu}{2}[1 - \gamma\sigma_1^s + \alpha(\gamma - \sigma_1^s)\sigma_2^s] \\ w_2(\sigma_2^s) = \frac{\nu}{2}[1 - \gamma\sigma_2^s + \alpha(\gamma - \sigma_2^s)\sigma_1^s] \end{cases} \tag{A5}$$

For $n \geq 3$,

$$\begin{cases} w_n(\sigma_1^s) = \frac{\nu}{2}[1 - \gamma\sigma_1^s + \alpha(\gamma - \sigma_1^s)\sigma_2^s] \\ w_n(\sigma_n^s) = \frac{\nu}{2}[1 - \gamma\sigma_n^s + \alpha(\gamma - \sigma_n^s)\sigma_{n-1}^s] \\ w_n(\sigma_i^s) = \frac{\nu}{2}[1 - \gamma\sigma_i^s + \alpha_1(\gamma - \sigma_i^s)(\sigma_{i-1}^s + \sigma_{i+1}^s)] \end{cases} \tag{A6}$$

Among them, $i = 2, \cdots n - 1$, $\alpha \equiv \tanh(\varpi)$, $\alpha_1 \equiv \frac{1}{2}\tanh(2\varpi)$.

The compact form of Eqs.A4-6 is,

$$w_n(\sigma_i^s) = \frac{\nu}{2}[1 - \gamma\sigma_i^s + \beta_i(\gamma - \sigma_i^s)(\sigma_{i-1}^s + \sigma_{i+1}^s)] \tag{A7}$$

where $i = 1, \cdots n$.

Appendix B: Mean-field of PSSs

The mean-field of PSSs have two parts. One is the mean-field approximation of strong to weak bonds, and the other is the effective interface-effect due to the interfaces between the groups of PSs and PS-vacancies, the RISF and RIEF.

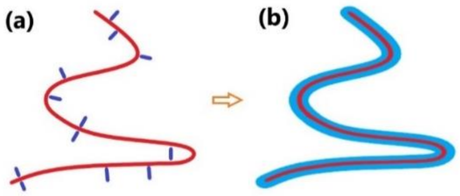

Fig.B1 Schematic plots of an n-g-PSS and its g randomly distributed strong bonds with the interaction strength being J in the 3D-ERSIM (a), and the averaged $4n$ uniform weak bonds of the strength $\frac{g}{4n}J$ (b).

Mean-field of strong to weak bonds. This part contains that the g randomly distributed strong bonds of n-g-PSSs with the interaction strength being J in the 3D-ERSIM (Fig.1d and Fig.B1a) are averaged to the $4n$ uniform weak bonds of the strength $\frac{g}{4n}J$ (Fig.B1b), and the Weiss-type[58,59] mean-field acting on each PS in the n-g-PSSs is $J\frac{g}{n}\eta_n^g$.

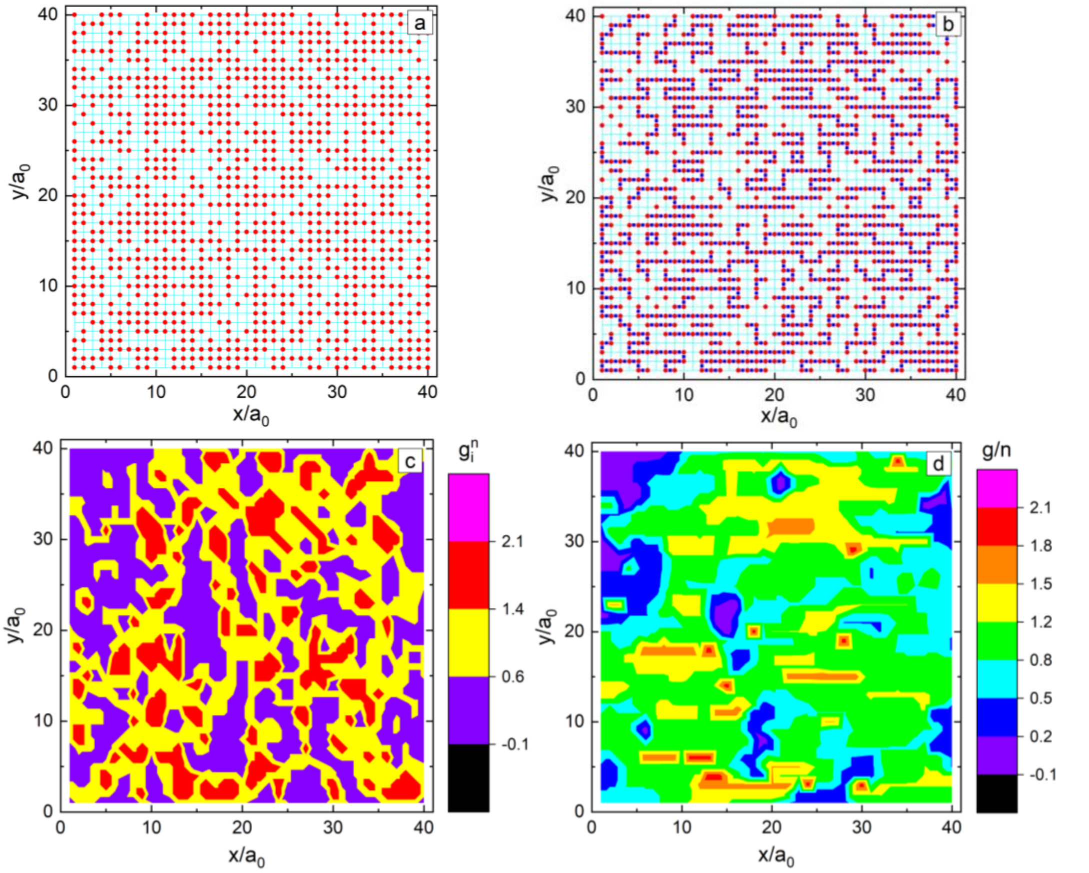

Fig.B2 (a) Simulated PS distribution in the x-y-plane (40×40 lattice points) of 2D-RSIM for $\phi = 1/3$. Cyan lines and red solid circles show the crystal lattice and PSs, respectively, while the unlabeled lattice points are PS-vacancies; (b) Connected PSSs in the x-y plane. The blue solid squares indicate the intra-string interaction bonds; (c) Surface plot of the spatial distribution of the inter-string bond number (g_i^n) of the ith-PS in an n-g-PSS; and (d) Surface plot of the average inter-string bond number (g/n) of n-g-PSSs.

In order to check the reliability of the above mean-field, the spatial distribution of the inter-string bond number (g_i^n) of the ith-PS in an n-g-PSS and the corresponding g/n in the x-y-plane (40×40 lattice points) of 2D-RSIM of $\phi = 1/3$ are shown in Fig.B2. It could be seen that: (i) The regions of $g_i^n = 2$ form individual clusters, and they mainly exist in the regions of $g_i^n = 1$; (ii) The regions of $g_i^n = 1$ and 0 are mutually embedded mosaic structures; and (iii) Although the distribution of different g/n regions are more flat, the overall characteristic is nearly same as that of g_i^n, which confirms the reliability of the mean-field.

Effective interface-effect. Ferdinand and Fisher[64] exactly calculated the specific-heat of finite 2D-Ising-model containing $n \times n$ spins. By comparing with the infinite system, it is found that: (i) The boundary effect of the finite system causes the dispersion of the specific-heat peak and the decrease of the peak temperature, i.e. the phase-transition temperature; and (ii) The influence is proportional to $1/n$

approximately. For 3D-RSIM, the interfaces between the PS and PS-vacancy groups will inevitably lead to the interface-effect, i.e. the dispersion of the phase-transition[64]. In this paper, we take this effect being proportional to $1/n^d$, where d is the interface-effect factor that needs further studies.

At present, there are two schemes to introduce the interface-effect in the Weiss mean-field method[58,59]: (i) Introduce an internal field unrelated to the order parameter (η); and (ii) Modify the mean-field proportional to the fractional one (η^h), where $0 < h < 1$. This article chooses the first scheme, and the internal field is taken as $J\frac{g}{n}\frac{f_d}{n^d}$, where f_d is the geometric factor of the interfaces ($f_d = 1$ is used herein). In addition, the interface-effect corrects the Weiss-type mean-field from $J\frac{g}{n}\eta_n^g$ to $J\frac{g}{n}\left(1-\frac{1}{n^d}\right)\eta_n^g$.

Therefore, with considering the effect of interfaces between the PS and PS-vacancy groups, the mean-field of the nearest-neighbor PSs to an n-g-PSS can be expressed as $J\frac{g}{n}\left[\left(1-\frac{1}{n^d}\right)\eta_n^g+\frac{1}{n^d}\right]$, and the corresponding Hamiltonian is $-J\frac{g}{n}\left[\left(1-\frac{1}{n^d}\right)\eta_n^g+\frac{1}{n^d}\right]\sum_{i=1}^{n}\sigma_i^s$.

Moreover, considering the fact that: (i) For heterovalent (PMN and $Sr_xBa_{1-x}Nb_2O_6$) and the isovalent (BZ_xT_{1-x}) RFEs have the same characteristics of relaxor-ferroelectricity[31-36,48-51], so the influence of E_i to the relaxor-ferroelectricity is the secondary compared with the primary 3D-RSIM (Eq.1a) as pointed by Kleemann et al.[4]; and (ii) An uniform static electric field can make a normal-ferroelectric phase-transition to a diffuse one[58,59], so, as an approximation, we take the influence of E_i as a factor (b_e) of effective interface-effect. In view of the similarity of RISF to RIEF[4], the influence of S_i can also be treated as another factor (b_s) of the effect.

Thus, the total effective interface-effect factor (b) is the sum of d, b_e, and b_s, i.e. $b = d + b_e + b_s$, and the corresponding Hamiltonian (H_{inter}^{ng}) that contains the inter-string interaction of an n-g-PSS, the RISF and RIEF can approximately be expressed as,

$$H_{inter}^{ng} = -J\frac{g}{n}\left[\left(1-\frac{1}{n^b}\right)\eta_n^g+\frac{1}{n^b}\right]\sum_{i=1}^{n}\sigma_i^s \tag{B1}$$

Appendix C: Calculation of Z_n^g and Q_n^g

The partition function (Z_n^g) of n-g-PSSs corresponding to H_n^g (Eq.2b) is,

$$Z_n^g \equiv \sum_{\sigma_1^s,\cdots\sigma_n^s}\exp\left[\varpi\sum_{i=1}^{n-1}\sigma_i^s\sigma_{i+1}^s+\theta\sum_{j=1}^{n}\sigma_j^s\right] \tag{C1}$$

From $\exp(\varpi\sigma_i^s\sigma_{i+1}^s) = (1+\alpha\sigma_i^s\sigma_{i+1}^s)\cosh(\varpi)$ and $\exp(\theta\sigma_j^s) = (1+\gamma\sigma_j^s)\cosh(\theta)$, we obtain,

$$Z_n^g = Q_n^g\cosh^{n-1}(\varpi)\cosh^n(\theta) \tag{C2}$$

where,

$$Q_n^g \equiv \sum_{\sigma_1^s,\cdots\sigma_n^s}\prod_{i=1}^{n-1}(1+\alpha\sigma_i^s\sigma_{i+1}^s)\prod_{j=1}^{n}(1+\gamma\sigma_j^s) \tag{C3}$$

By introducing a variable,

$$Y_n^g \equiv \sum_{\sigma_1^s,\cdots\sigma_n^s}\prod_{i=1}^{n-1}(1+\alpha\sigma_i^s\sigma_{i+1}^s)\prod_{j=1}^{n}(1+\gamma\sigma_j^s)\,\sigma_n^s \tag{C4}$$

(Obviously, $Q_0^g = 1$, $Y_0^g = 0$, $Q_1^g = 2$ and $Y_1^g = 2\gamma$), the following recurrence formulas are derived from Eqs.C3-4,

$$\begin{cases} Q_n^g = 2\left(Q_{n-1}^g+\alpha\gamma Y_{n-1}^g\right) \\ Y_n^g = 2\left(\gamma Q_{n-1}^g+\alpha Y_{n-1}^g\right) \end{cases} \tag{C5}$$

Let,

$$A_n^g \equiv Q_n^g + BY_n^g \tag{C6}$$

to make,

$$A_n^g = DA_{n-1}^g \tag{C7}$$

where B and D are pending constants, and from Eqs.C5-7, we obtain,

$$D\left(Q_n^g+BY_n^g\right) = 2\left[(1+\gamma B)Q_n^g+\alpha(\gamma+B)Y_n^g\right] \tag{C8}$$

and,

$$\begin{cases} 2(1+\gamma B) = D \\ 2\alpha(\gamma+B) = BD \end{cases} \tag{C9}$$

So, the two values of B and D are,

$$\begin{cases} B_1 = \dfrac{\alpha - 1 + \Omega}{2\gamma} \\ B_2 = \dfrac{\alpha - 1 - \Omega}{2\gamma} \end{cases} \tag{C10}$$

$$\begin{cases} D_1 = 1 + \alpha + \Omega \\ D_2 = 1 + \alpha - \Omega \end{cases} \tag{C11}$$

where $\Omega \equiv \sqrt{(1-\alpha)^2 + 4\alpha\gamma^2}$.

The corresponding two values of A_n^g are,

$$\begin{cases} A_{n1}^g = {D_1}^n \\ A_{n2}^g = {D_2}^n \end{cases} \tag{C12}$$

By Eqs.C6 and C12, it is obtained,

$$\begin{cases} Q_n^g + B_1 Y_n^g = {D_1}^n \\ Q_n^g + B_2 Y_n^g = {D_2}^n \end{cases} \tag{C13}$$

Therefore,

$$\begin{cases} Q_n^g = G_1 {D_2}^n + G_2 {D_1}^n \\ Y_n^g = \dfrac{\gamma}{\Omega}\left({D_1}^n - {D_2}^n\right) \end{cases} \tag{C14}$$

where $G_1 \equiv \frac{1}{2} - \frac{1-\alpha}{2\Omega}$ and $G_2 \equiv \frac{1}{2} + \frac{1-\alpha}{2\Omega}$.

Appendix D: Calculation of s_{nk}^g

According to H_n^g (Eq.2b), the expectation value (s_{nk}^g) of σ_k in n-g-PSSs is,

$$s_{nk}^g \equiv \frac{1}{Z_n^g} \sum_{\sigma_1^s,\cdots\sigma_n^s} \sigma_k^s \exp\left[\varpi \sum_{i=1}^{n-1} \sigma_i^s \sigma_{i+1}^s + \theta \sum_{j=1}^{n} \sigma_j^s\right] \tag{D1}$$

and,

$$s_{nk}^g = \frac{1}{Q_n^g} \sum_{\sigma_1^s,\cdots\sigma_n^s} \prod_{i=1}^{n-1} \prod_{j=1}^{n} \left(1 + \alpha\sigma_i^s\sigma_{i+1}^s\right)\left(1 + \gamma\sigma_j^s\right) \sigma_k^s \tag{D2}$$

By $(1 + \alpha\sigma_k^s\sigma_{k+1}^s)\sigma_k^s = \sigma_k^s + \alpha\sigma_{k+1}^s$, we get,

$$s_{nk}^g = \frac{1}{Q_n^g} \sum_{\sigma_1^s,\cdots\sigma_k^s} \prod_{i=1}^{k-1} \prod_{j=1}^{k} \left(1 + \alpha\sigma_i^s\sigma_{i+1}^s\right)\left(1 + \gamma\sigma_j^s\right)\sigma_k^s \sum_{\sigma_{k+1}^s,\cdots\sigma_n^s} \prod_{i=k+1}^{n-1} \prod_{j=k+1}^{n} \left(1 + \alpha\sigma_i^s\sigma_{i+1}^s\right)\left(1 + \gamma\sigma_j^s\right)$$

$$+ \frac{\alpha}{Q_n^g} \sum_{\sigma_1^s,\cdots\sigma_k^s} \prod_{i=1}^{k-1} \prod_{j=1}^{k} \left(1 + \alpha\sigma_i^s\sigma_{i+1}^s\right)\left(1 + \gamma\sigma_j^s\right) \sum_{\sigma_{k+1}^s,\cdots\sigma_n^s} \prod_{i=k+1}^{n-1} \prod_{j=k+1}^{n} \left(1 + \alpha\sigma_i^s\sigma_{i+1}^s\right)\left(1 + \gamma\sigma_j^s\right)\sigma_{k+1}^s$$

i.e.,

$$s_{nk}^g = \frac{1}{Q_n^g}\left(Y_k^g Q_{n-k}^g + \alpha Q_k^g Y_{n-k}^g\right) \tag{D3}$$

and,

$$s_{nk}^g = s_{nn-k+1}^g \tag{D4}$$

Appendix E: Calculation of χ_s^{ng}

Along the direction of PSs in 3D-ERSIM, an external electric field (E) small enough is loaded, and the Hamiltonian of n-g-PSSs (Eq.2b) becomes,

$$H_{nE}^g = -J \sum_{i=1}^{n-1} \sigma_i^s \sigma_{i+1}^s - \left\{J \frac{g}{n}\left[\left(1 - \frac{1}{n^b}\right)\eta_n^{gE} + \frac{1}{n^b}\right] + \mu E\right\} \sum_{j=1}^{n} \sigma_j^s \tag{E1}$$

The corresponding partition function (Z_{nE}^g) and order parameter (η_n^{gE}) are, respectively,

$$Z_{nE}^g \equiv \sum_{\sigma_1^s,\cdots\sigma_n^s} \exp\left[\varpi \sum_{i=1}^{n-1} \sigma_i^s \sigma_{i+1}^s + \theta^E \sum_{j=1}^{n} \sigma_j^s\right] \tag{E2}$$

$$\eta_n^{gE} = \frac{1}{n Z_{nE}^g} \frac{\partial Z_{nE}^g}{\partial \theta^E} \tag{E3}$$

where $\theta^E \equiv \theta + \frac{\mu E}{k_B T}$. Since Z_{nE}^g is an even function about E, $Z_{nE}^g = Z_n^g + O(E^2)$ as $E \to 0$。

By Eqs.E1-3, the static permittivity (χ_s^{ng}) of n-g-PSSs in thermal equilibrium is,

$$\chi_s^{ng} \equiv \frac{n\mu}{\varepsilon_0}\frac{\partial \eta_n^{gE}}{\partial E} = \frac{n\mu}{\varepsilon_0}\frac{\partial \eta_n^{gE}}{\partial \theta^E}\frac{\partial\theta^E}{\partial E} = \frac{\partial \eta_n^{gE}}{\partial\theta^E}\left[\frac{n\mu}{\varepsilon_0}\frac{\partial\eta_n^{gE}}{\partial E}\frac{A_n^g}{T} + \frac{n}{N_0}\frac{C_w}{T}\right] = \aleph_n^g\left(\frac{A_n^g}{T}\chi_s^{n,g} + \frac{n}{N_0}\frac{C_w}{T}\right)$$

and we get,

$$\chi_s^{ng} = \frac{nC_w}{N_0}\frac{\aleph_n^g}{T-\aleph_n^g A_n^g} \tag{E4}$$

where $\aleph_n^g \equiv \left.\frac{\partial\eta_n^{gE}}{\partial\theta^E}\right|_{E\to0} = \frac{\partial\eta_{ne}^g}{\partial\theta_e} = \left\{1-\gamma^2+\frac{1}{nQ_n^g}\left[\frac{\partial^2 Q_n^g}{\partial\theta^2} - \frac{1}{Q_n^g}\left(\frac{\partial Q_n^g}{\partial\theta}\right)^2\right]\right\}_{\gamma=\gamma_e,\theta=\theta_e}$.

Appendix F: Calculation of ζ_{nk}^g

According to its definition of ζ_{nk}^g,

$$\zeta_{nk}^g \equiv \frac{1}{Z_n^g}\sum_{\sigma_1^s,\cdots\sigma_n^s}\sigma_k^s\sigma_{k+1}^s\exp\left[\varpi\sum_{i=1}^{n-1}\sigma_i^s\sigma_{i+1}^s + \theta\sum_{j=1}^{n}\sigma_j^s\right] \tag{F1}$$

and we get,

$$\zeta_{nk}^g = \frac{1}{Q_n^g}\sum_{\sigma_1^s,\cdots\sigma_n^s}\prod_{i=1}^{n-1}(1+\alpha\sigma_i^s\sigma_{i+1}^s)\prod_{j=1}^{n}(1+\gamma\sigma_j^s)\,\sigma_k^s\sigma_{k+1}^s \tag{F2}$$

By $(1+\alpha\sigma_k^s\sigma_{k+1}^s)\sigma_k^s\sigma_{k+1}^s = \alpha + \sigma_k^s\sigma_{k+1}^s$, we obtain,

$$\zeta_{nk}^g = \frac{1}{Q_n^g}\left[\alpha\sum_{\sigma_1^s,\cdots\sigma_k^s}\prod_{i=1}^{k-1}(1+\alpha\sigma_i^s\sigma_{i+1}^s)\prod_{j=1}^{k}(1+\gamma\sigma_j^s)\sum_{\sigma_{k+1}^s,\cdots\sigma_n^s}\prod_{i=k+1}^{n-1}(1+\alpha\sigma_i^s\sigma_{i+1}^s)\prod_{j=k+1}^{n}(1+\gamma\sigma_j^s)\right.$$
$$+\sum_{\sigma_1^s,\cdots\sigma_k^s}\prod_{i=1}^{k-1}(1+\alpha\sigma_i^s\sigma_{i+1}^s)\prod_{j=1}^{k}(1+\gamma\sigma_j^s)\,\sigma_k^s\sum_{\sigma_{k+1}^s,\cdots\sigma_n}\prod_{i=k+1}^{n-1}(1+\alpha\sigma_i^s\sigma_{i+1}^s)\prod_{j=k+1}^{n}(1$$
$$\left.+\gamma\sigma_j^s)\,\sigma_{k+1}^s\right]$$

i.e.,

$$\zeta_{nk}^g = \frac{1}{Q_n^g}\left(Y_k^g Y_{n-k}^g + \alpha Q_k^g Q_{n-k}^g\right) \tag{F3}$$

Obviously,

$$\zeta_{nk}^g = \zeta_{nn-k}^g \tag{F4}$$

Appendix G: Relaxation equation of s_{nk}^g

As shown in Eq.2d, the Glauber transition probability of n-g-PSSs is slightly different to 1D-infinite spin chains[29]. For the sake of clarity, the relaxation equation of s_{nk}^g in n-g-PSSs is derived here.

In an n-g-PSS, the probability that the n PSs take specific values $(\sigma_1^s,\cdots\sigma_n^s)$ is,

$$p(\sigma_1^s,\cdots\sigma_n^s) = \frac{1}{Z_n^g}\exp\left[\varpi\sum_{i=1}^{n-1}\sigma_i^s\sigma_{i+1}^s + \theta\sum_{j=1}^{n}\sigma_j^s\right] \tag{G1}$$

Then,

$$\frac{dp(\sigma_1^s,\cdots\sigma_n^s)}{dt} = \sum_i^n w_n(-\sigma_i^s)p(\sigma_1^s,\cdots-\sigma_i^s,\cdots\sigma_n^s)$$
$$-\sum_j^n w_n(\sigma_i^s)p(\sigma_1^s,\cdots\sigma_i^s,\cdots\sigma_n^s) \tag{G2}$$

and,

$$s_{nk}^g = \sum_{\sigma_1^s,\cdots\sigma_n^s}\sigma_k^s p(\sigma_1^s,\cdots\sigma_n^s) \tag{G3}$$

By multiplying both sides of Eq.G2 by σ_k^s and calculating the sum of $\sigma_1^s,\cdots\sigma_n^s$, we obtain,

$$\frac{ds_{nk}^{g}}{dt} = \sum_{\sigma_1^s,\cdots\sigma_n^s} \sigma_k^s \left[\sum_{i}^{n} w_n(-\sigma_i^s) p(\sigma_1^s, \cdots - \sigma_i^s, \cdots \sigma_n^s) \right.$$

$$\left. - \sum_{j}^{n} w_n(\sigma_i^s) p(\sigma_1^s, \cdots \sigma_i^s, \cdots \sigma_n^s) \right] \qquad \text{(G4)}$$

In the right side of Eq.G4, the terms of $i = k$ can be simplified as,

$$\sum_{\sigma_1^s,\cdots\sigma_n^s} \sigma_k^s [w_n(-\sigma_k^s) p(\sigma_1^s, \cdots - \sigma_k^s, \cdots \sigma_n^s) - w_n(\sigma_k^s) p(\sigma_1^s, \cdots \sigma_k^s, \cdots \sigma_n^s)]$$

$$= -2 \sum_{\sigma_1^s,\cdots\sigma_n^s} \sigma_k^s w_n(\sigma_k^s) p(\sigma_1^s, \cdots \sigma_k, \cdots \sigma_n^s) \qquad \text{(G5)}$$

and by,

$$\sum_{\sigma_i^s} [w_n(-\sigma_i^s) p(\sigma_1^s, \cdots - \sigma_i^s, \cdots \sigma_n^s) - w_n(\sigma_i^s) p(\sigma_1^s, \cdots \sigma_i^s, \cdots \sigma_n^s)] = 0$$

we obtain that the terms of $i \neq k$ in the right side of Eq.G4 are all equal zero. So,

$$\frac{ds_{nk}^{g}}{dt} = -2 \sum_{\sigma_1^s,\cdots\sigma_n^s} \sigma_k^s w_n(\sigma_k^s) p(\sigma_1^s, \cdots \sigma_n^s) \qquad \text{(G6)}$$

By substituting the expression of $w_n(\sigma_k^s)$ (Eq.2d) into Eq.G6, the relaxation equation of s_{nk}^{g} is:

For $n = 1$,

$$\frac{1}{\nu}\frac{ds_{11}^{g}}{dt} = -s_{11}^{g} + \gamma \qquad \text{(G7)}$$

For $n = 2$,

$$\begin{cases} \frac{1}{\nu}\frac{ds_{21}^{g}}{dt} = -s_{21}^{g} + \alpha s_{22}^{g} + \gamma(1 - \alpha\zeta_{21}^{g}) \\ \frac{1}{\nu}\frac{ds_{22}^{g}}{dt} = -s_{22}^{g} + \alpha s_{21}^{g} + \gamma(1 - \alpha\zeta_{21}^{g}) \end{cases} \qquad \text{(G8)}$$

For $n \geq 3$,

$$\begin{cases} \frac{1}{\nu}\frac{ds_{n1}^{g}}{dt} = -s_{n1}^{g} + \alpha s_{n2}^{g} + \gamma(1 - \alpha\zeta_{n1}^{g}) \\ \frac{1}{\nu}\frac{ds_{nn}^{g}}{dt} = -s_{n1}^{g} + \alpha s_{nn-1}^{g} + \gamma(1 - \alpha\zeta_{nn-1}^{g}) \\ \frac{1}{\nu}\frac{ds_{nk}^{g}}{dt} = -s_{nk}^{g} + \alpha_1(s_{nk-1}^{g} + s_{nk+1}^{g}) + \gamma[1 - \alpha_1(\zeta_{nk-1}^{g} + \zeta_{nk}^{g})] \end{cases} \qquad \text{(G9)}$$

where $k = 2, \cdots n - 1$.

The more compact form of Eqs.G7-9 is,

$$\frac{1}{\nu}\frac{ds_{ni}^{g}}{dt} = -s_{ni}^{g} + \beta_i(s_{ni-1}^{g} + s_{ni+1}^{g}) + \gamma[1 - \beta_i(\zeta_{ni-1}^{g} + \zeta_{ni}^{g})] \qquad \text{(G10)}$$

where $i = 1, \cdots n$.

Appendix H: Relaxation equation of δ_{nk}^{g}

In this appendix, we derive the relaxation equations of δ_{nk}^{g} $(k = 1, \cdots n)$ vs t according to Eq.G10, and give their general solutions.

Relaxation equation of δ_{11}^{g}. By substituting $\eta_{1e}^{g} = s_{11}^{ge}$, $s_{11}^{g} = s_{11}^{ge} + \delta_{11}^{g}$, $s_{11}^{ge} = \gamma_e$, $\gamma = \gamma_e + a_1^g \delta_{11}^{g}$, $a_1^g \equiv \frac{\partial\gamma_e}{\partial s_{11}^{ge}} = \frac{\partial\gamma_e}{\partial\theta_e}\frac{\partial\theta_e}{\partial\eta_{1e}^{g}}\frac{\partial\eta_{1e}^{g}}{\partial s_{11}^{ge}}$, $\frac{\partial\gamma_e}{\partial\theta_e} = 1 - {\gamma_e}^2$, $\frac{\partial\theta_e}{\partial\eta_{1e}^{g}} = \frac{A_1^g}{T}$, $A_1^g \equiv \left(1 - \frac{1}{1^b}\right)\Theta_1^g = 0$, and $\frac{\partial\eta_{1e}^{g}}{\partial s_{11}^{ge}} = 1$ into Eq.G7, we obtain,

$$\frac{1}{\nu}\frac{d\delta_{11}^{g}}{dt} = -\delta_{11}^{g} \qquad \text{(H1)}$$

The general solution of this linear homogeneous differential equation is,

$$\delta_{11}^{g} = \delta_{11}^{g}(0)\exp\left(-\frac{t}{\tau_{11}^{g}}\right) \qquad \text{(H2)}$$

where $\delta_{11}^{g}(0)$ is the value of δ_{11}^{g} at $t = 0$, and,

$$\tau_{11}^{g} = \frac{1}{\nu} \tag{H3}$$

Relaxation equation of δ_{2k}^{g}. By substituting $s_{2k}^{g} = s_{2k}^{ge} + \delta_{2k}^{g}$; $\gamma = \gamma_{e} + a_{2}^{g}\left(\delta_{21}^{g} + \delta_{22}^{g}\right)$, $a_{2}^{g} \equiv \frac{\partial \gamma_{e}}{\partial s_{2k}^{ge}} = \frac{\partial \gamma_{e}}{\partial \theta_{e}} \frac{\partial \theta_{e}}{\partial \eta_{2e}^{g}} \frac{\partial \eta_{2e}^{g}}{\partial s_{2k}^{ge}}$, $\frac{\partial \theta_{e}}{\partial \eta_{2e}^{g}} = \frac{A_{2}^{g}}{T}$, $A_{2}^{g} \equiv \left(1 - \frac{1}{2^{b}}\right) \Theta_{2}^{g}$, $\Theta_{2}^{g} = \frac{g}{2} \Theta_{J}$, $\frac{\partial \eta_{2e}^{g}}{\partial s_{2k}^{ge}} = \frac{1}{2}$, $\zeta_{21}^{g} = \zeta_{21}^{ge} + b_{21}^{g} a_{2}^{g}\left(\delta_{21}^{g} + \delta_{22}^{g}\right)$, $b_{21}^{g} \equiv \frac{\partial \zeta_{21}^{ge}}{\partial \gamma_{e}}$, and $\zeta_{21}^{ge} = \zeta_{21}^{g}\big|_{\gamma=\gamma_{e}}$ into Eq.G8, we get,

$$\frac{1}{\nu}\frac{d}{dt}\begin{bmatrix}\delta_{21}^{g}\\ \delta_{22}^{g}\end{bmatrix} = -[M]_{22}\begin{bmatrix}\delta_{21}^{g}\\ \delta_{22}^{g}\end{bmatrix} \tag{H4}$$

where $[M]_{22} = \begin{bmatrix}1 & -\alpha\\ -\alpha & 1\end{bmatrix} - \begin{bmatrix}d_{21}^{g} & d_{21}^{g}\\ d_{22}^{g} & d_{22}^{g}\end{bmatrix}$ and $d_{21}^{g} = d_{22}^{g} \equiv a_{2}^{g}\left[1 - \alpha\left(\zeta_{21}^{ge} + \gamma_{e} b_{21}^{g}\right)\right]$.

The general solution of the linear homogeneous differential equations (Eq.H4) is,

$$\begin{bmatrix}\delta_{21}^{g}\\ \delta_{22}^{g}\end{bmatrix} = e^{-\lambda_{2}^{g}\nu t}\begin{bmatrix}V_{21}^{g}\\ V_{22}^{g}\end{bmatrix} \tag{H5}$$

where $[V_{21}^{g}, V_{22}^{g}]$ is the eigenvector of λ_{2}^{g}.

By substituting Eq.H5 into Eq.H4, it is obtained,

$$\lambda_{2}^{g}\begin{bmatrix}V_{21}^{g}\\ V_{22}^{g}\end{bmatrix} = \begin{bmatrix}M_{11} & M_{12}\\ M_{21} & M_{22}\end{bmatrix}\begin{bmatrix}V_{21}^{g}\\ V_{22}^{g}\end{bmatrix} \tag{H6}$$

The condition that these homogeneous linear equations have non-zero $[V_{21}^{g}, V_{22}^{g}]$ is,

$$\det\begin{vmatrix}\lambda_{2}^{g} - 1 + d_{21}^{g} & \alpha + d_{21}^{g}\\ \alpha + d_{21}^{g} & \lambda_{2}^{g} - 1 + d_{21}^{g}\end{vmatrix} = 0 \tag{H7}$$

where $\det|X|$ is the determinant of matrix X.

Eq.H7 is the characteristic equation of λ_{2}^{g}, and the two eigenvalues of λ_{2}^{g} are,

$$\begin{cases}\lambda_{21}^{g} = 1 + \alpha\\ \lambda_{22}^{g} = 1 - \alpha - 2d_{21}^{g}\end{cases} \tag{H8}$$

For $\lambda_{21}^{g} = 1 + \alpha$, the corresponding eigenvector $[V_{21}^{g1}, V_{22}^{g1}]$ satisfies the following equations,

$$\lambda_{21}^{g}\begin{bmatrix}V_{21}^{g1}\\ V_{22}^{g1}\end{bmatrix} = \begin{bmatrix}1 - d_{21}^{g} & -\alpha - d_{21}^{g}\\ -\alpha - d_{2}^{g1} & 1 - d_{21}^{g}\end{bmatrix}\begin{bmatrix}V_{21}^{g1}\\ V_{22}^{g1}\end{bmatrix} \tag{H9}$$

and,

$$\begin{bmatrix}V_{21}^{g1}\\ V_{22}^{g1}\end{bmatrix} = V_{22}^{g1}\begin{bmatrix}-1\\ 1\end{bmatrix} \tag{H10}$$

For $\lambda_{22}^{g} = 1 - \alpha - 2d_{21}^{g}$, the corresponding eigenvector $[V_{21}^{g2}, V_{22}^{g2}]$ obeys the following equations,

$$\lambda_{22}^{g}\begin{bmatrix}V_{21}^{g2}\\ V_{22}^{g2}\end{bmatrix} = \begin{bmatrix}1 - d_{21}^{g} & -\alpha - d_{21}^{g}\\ -\alpha - d_{21}^{g} & 1 - d_{21}^{g}\end{bmatrix}\begin{bmatrix}V_{21}^{g2}\\ V_{22}^{g2}\end{bmatrix} \tag{H11}$$

and,

$$\begin{bmatrix}V_{21}^{g2}\\ V_{22}^{g2}\end{bmatrix} = V_{22}^{g2}\begin{bmatrix}1\\ 1\end{bmatrix} \tag{H12}$$

$[-1,1]$ and $[1,1]$ are, respectively, the reduced eigenvectors of λ_{21}^{g} and λ_{22}^{g}, and we get,

$$\begin{bmatrix}\delta_{21}^{g}\\ \delta_{22}^{g}\end{bmatrix} = V_{22}^{g1}\begin{bmatrix}-1\\ 1\end{bmatrix}\exp\left(\frac{t}{\tau_{21}^{g}}\right) + V_{22}^{g2}\begin{bmatrix}1\\ 1\end{bmatrix}\exp\left(\frac{t}{\tau_{22}^{g}}\right) \tag{H13}$$

as well as,

$$\tau_{2k}^{g} \equiv \frac{1}{\nu\lambda_{2k}^{g}}, k = 1,2 \tag{H14}$$

Relaxation equation of δ_{nk}^{g} $(n \geq 3)$. By substituting $s_{nk}^{g} = s_{nk}^{ge} + \delta_{nk}^{g}$, $\gamma = \gamma_{e} + a_{n}^{g}\sum_{k=1}^{n}\delta_{nk}^{g}$, $a_{n}^{g} \equiv \frac{\partial \gamma_{e}}{\partial s_{nk}^{ge}} = \frac{\partial \gamma_{e}}{\partial \theta_{e}} \frac{\partial \theta_{e}}{\partial \eta_{ne}^{g}} \frac{\partial \eta_{ne}^{g}}{\partial s_{nk}^{ge}}$, $\frac{\partial \theta_{e}}{\partial \eta_{ne}^{g}} = \frac{A_{n}^{g}}{T}$, $\frac{\partial \eta_{ne}^{g}}{\partial s_{nk}^{ge}} = \frac{1}{n}$, $A_{n}^{g} \equiv \left(1 - \frac{1}{n^{b}}\right)\Theta_{n}^{g}$, $\zeta_{nk}^{g} = \zeta_{nk}^{ge} + b_{nk}^{g} a_{n}^{g}\sum_{k=1}^{n}\delta_{nk}^{g}$, $b_{nk}^{g} \equiv \frac{\partial \zeta_{nk}^{ge}}{\partial \gamma_{e}}$, $\frac{\partial \zeta_{nk}^{ge}}{\partial s_{nk}^{ge}} = \frac{\partial \zeta_{nk}^{ge}}{\partial \gamma_{e}} \frac{\partial \gamma_{e}}{\partial s_{nk}^{ge}} = b_{nk}^{g} a_{n}^{g}$, and $\zeta_{nk}^{ge} = \zeta_{nk}^{g}\big|_{\gamma=\gamma_{e}}$ into Eq.G9, we obtain,

$$\frac{1}{\nu}\frac{d}{dt}\begin{bmatrix}\delta_{n1}^{g}\\ \vdots\\ \delta_{nn}^{g}\end{bmatrix} = -\begin{bmatrix}M_{11} & \cdots & M_{1n}\\ \vdots & \ddots & \vdots\\ M_{n1} & \cdots & M_{nn}\end{bmatrix}\begin{bmatrix}\delta_{n1}^{g}\\ \vdots\\ \delta_{nn}^{g}\end{bmatrix} \tag{H15}$$

where the square matrix is,

$$[M]_{nn} = -\begin{bmatrix} -1 & \alpha & 0 & & & & \\ \alpha_1 & -1 & \alpha_1 & \cdots & & 0 & \\ 0 & \alpha_1 & -1 & & & & \\ & \vdots & & \ddots & & \vdots & \\ & & & & -1 & \alpha_1 & 0 \\ & 0 & & \cdots & \alpha_1 & -1 & \alpha_1 \\ & & & & 0 & \alpha & -1 \end{bmatrix}$$

$$-\begin{bmatrix} d_{n1}^g & d_{n1}^g & \cdots & d_{n1}^g & d_{n1}^g \\ d_{n2}^g & d_{n2}^g & & d_{n2}^g & d_{n2}^g \\ \vdots & & \ddots & & \vdots \\ d_{nn-1}^g & d_{nn-1}^g & \cdots & d_{nn-1}^g & d_{nn-1}^g \\ d_{nn}^g & d_{nn}^g & & d_{nn}^g & d_{nn}^g \end{bmatrix} \tag{H16}$$

and,

$$\begin{cases} d_{n1}^g \equiv a_n^g\left[1 - \alpha\left(\zeta_{n1}^{ge} + \gamma_e b_{n1}^g\right)\right] \\ d_{nn}^g \equiv a_n^g\left[1 - \alpha\left(\zeta_{nn-1}^{ge} + \gamma_e b_{nn-1}^g\right)\right] \\ d_{nk}^g \equiv a_n^g\left[1 - \alpha_1\left(\zeta_{nk-1}^{ge} + \zeta_{nk}^{ge}\right) - \alpha_1\gamma_e\left(b_{nk-1}^g + b_{nk}^g\right)\right] \end{cases} \tag{H17}$$

where $k = 2, \cdots n - 1$.

The general solution of the linear homogeneous differential equations (Eq.H15) is,

$$\begin{bmatrix} \delta_{n1}^g \\ \vdots \\ \delta_{nn}^g \end{bmatrix} = e^{-\lambda_n^g v t} \begin{bmatrix} V_{n1}^g \\ \vdots \\ V_{nn}^g \end{bmatrix} \tag{H18}$$

where $[V_{n1}^g, \cdots V_{nn}^g]$ is the eigenvector of λ_n^g.

By substituting this general solution into Eq.H15, we obtain,

$$\lambda_n^g \begin{bmatrix} V_{n1}^g \\ \vdots \\ V_{nn}^g \end{bmatrix} = \begin{bmatrix} M_{11} & \cdots & M_{1n} \\ \vdots & \ddots & \vdots \\ M_{n1} & \cdots & M_{nn} \end{bmatrix} \begin{bmatrix} V_{n1}^g \\ \vdots \\ V_{nn}^g \end{bmatrix} \tag{H19}$$

The condition that the homogeneous linear equations (Eq.H19) have non-zero $[V_{n1}^g, \cdots V_{nn}^g]$ is,

$$\det\left|[M]_{nn} - \lambda_n^g [I]_{nn}\right| = 0 \tag{H20}$$

and this equation is called the characteristic equation of λ_n^g, where $[I]_{nn}$ is an n-order unit square matrix.

The n eigenvalues $(\lambda_{n1}^g, \cdots \lambda_{nn}^g)$ of $[M]_{nn}$ can be obtained from Eq.H20, and the eigenvector $[V_{n1}^{gk}, \cdots V_{nn}^{gk}]$ corresponding to λ_{nk}^g satisfies the following equations,

$$\lambda_{nk}^g \begin{bmatrix} V_{n1}^{gk} \\ \vdots \\ V_{nn}^{gk} \end{bmatrix} = \begin{bmatrix} M_{11} & \cdots & M_{1n} \\ \vdots & \ddots & \vdots \\ M_{n1} & \cdots & M_{nn} \end{bmatrix} \begin{bmatrix} V_{n1}^{gk} \\ \vdots \\ V_{nn}^{gk} \end{bmatrix} \tag{H21}$$

Since Eq.H21 is homogeneous linear equations of V_{ni}^{gk}, only $n - 1$ values in $[V_{n1}^{gk}, \cdots V_{nn}^{gk}]$ can be determined. By defining $v_{ni}^{gk} \equiv \frac{V_{ni}^{gk}}{V_{nn}^{gk}}, i = 1, \cdots n$, the reduced eigenvector $[v_{n1}^{gk}, \cdots 1]$ of λ_{nk}^g obeys the following equations,

$$\begin{bmatrix} M_{11} - \lambda_{nk}^g & \cdots & M_{1n-1} \\ \vdots & \ddots & \vdots \\ M_{n-11} & \cdots & M_{n-1n-1} - \lambda_{nk}^g \end{bmatrix} \begin{bmatrix} v_{n1}^{gk} \\ \vdots \\ v_{nn-1}^{gk} \end{bmatrix} = -\begin{bmatrix} M_{1n} \\ \vdots \\ M_{n-1n} \end{bmatrix} \tag{H22}$$

Therefore,

$$\begin{bmatrix} \delta_{n1}^g \\ \vdots \\ \delta_{nn}^g \end{bmatrix} = \sum_{k=1}^{n} V_{nn}^{gk} \begin{bmatrix} v_{n1}^{gk} \\ \vdots \\ 1 \end{bmatrix} \exp\left(\frac{t}{\tau_{nk}^g}\right) \tag{H23}$$

and,

$$\tau_{nk}^g \equiv \frac{1}{v\lambda_{nk}^g}, k = 1, \cdots n \tag{H24}$$

The physical meaning of the solution of Eqs.H2, H13, and H23 is that the coupling relaxation of δ_{nk}^{g} $(k = 1,\cdots n)$ is equivalent to n mutually independent spatial-relaxation-modes (SRMs), which have different relaxation times ($\tau_{nk}^{g}, k = 1,\cdots n$) (Eqs.H2, H7, and H20) and spatial distributions (v_{ni}^{gk}, $k, i = 1,\cdots n$) (Eqs.H10, H12, H21, and H22). Here, from short to long τ_{nk}^{g} $(k = 1,\cdots n)$, all the SRMs are referred as 1st-, ⋯ to nth-SRM, respectively.

$\nu\tau_{nk}^{g}$ $(k = 1,\cdots n)$ vs T for $b = 1.5$ and serial n and $g/n = 2$ are shown in Fig.H1. We can see that $\nu\tau_{nn}^{g}$ has a diffuse peak near T_p^{ng}, while $\nu\tau_{nk}^{g}$ $(k = 1,\cdots n-1)$ has not the peak, and $\nu\tau_{nn}^{g}$ increases again with decreasing T, until diverges at $T =$ 0K.

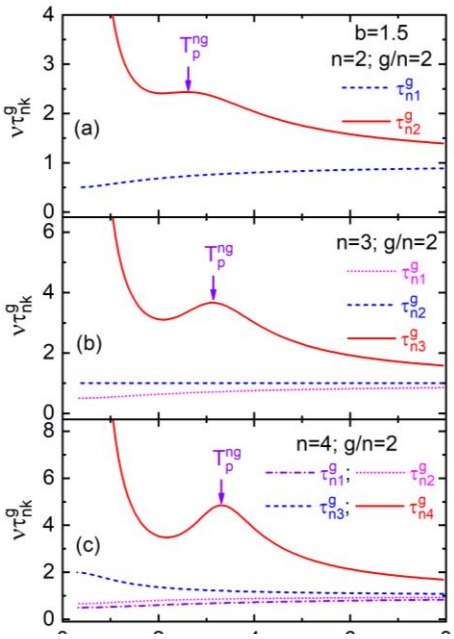

Fig.H1 $\nu\tau_{nk}^{g}$ $(k = 1,\cdots n)$ of n-g-PSSs with $b = 1.5$ vs T for $g/n = 2$ and $n = 2, 3, 4$. T_p^{ng} is the DPT temperature.

Fig.H2 shows v_{ni}^{gk} $(k = 1,\cdots n)$ of SRMs vs the PS position $(i = 1,\cdots n)$ in n-g-PSSs when $n = 4$, $b = 1.5$, $g/n = 3$, and serial T. Combining the exact results of $n = 1, 2$ (Eqs.H10, H12, and H22), we can conclude that the characteristic of v_{ni}^{gk} is similar to standing wave. Similarly, we also use the nodes of v_{ni}^{gk} $(k, i = 1,\cdots n)$, where v_{ni}^{gk} is equal to zero, or two neighboring v_{ni}^{gk} intersect at zero, and specifically, the nth-, ⋯ 1st-SRMs, respectively, have 0, ⋯ $n-1$ nodes, and the larger the number of nodes, the more inhomogeneous v_{ni}^{gk} is. For the nth-, n-2th- ⋯ SRMs whose node numbers are even, $v_{ni}^{gk} = v_{nn-i-1}^{gk}$ $(k = n, n-2\cdots)$, i.e. mirror symmetry along the PSSs. For the n-1th-, n-3th- ⋯ SRMs with odd number of nodes, $v_{ni}^{gk} = -v_{nn-i-1}^{gk}$ $(k = n-1, n-3\cdots)$, i.e. mirror anti-symmetry along the PSSs.

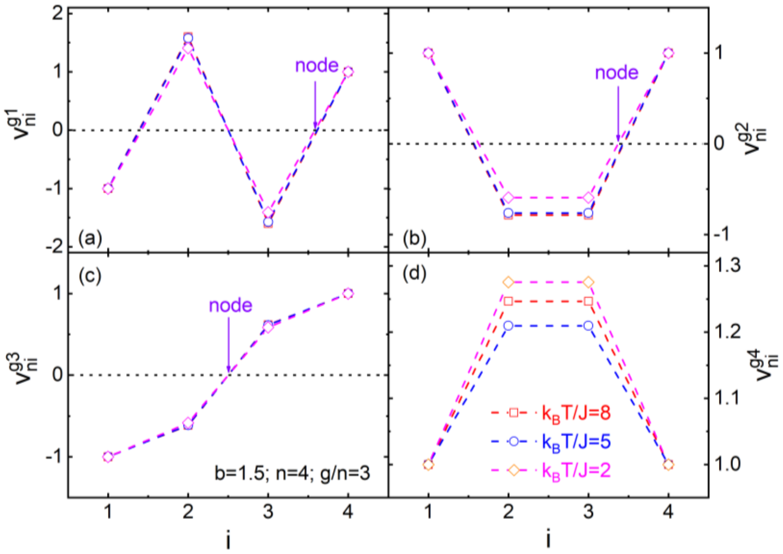

Fig.H2 v_{ni}^{gk} $(k = 1,\cdots n)$ of SRMs vs the PS position $(i = 1,\cdots n)$ in n-g-PSSs for $b = 1.5$, $n = 4$, and $g/n = 3$.

Appendix I: Complex permittivity of n-g-PSSs

After the following small enough and step-like external electric field (E) with t,

$$E = \begin{cases} E_0, t \le 0 \\ 0, \;\; t > 0 \end{cases} \tag{I1}$$

is applied to the 3D-ERSIGM along the PS direction, the Hamiltonian of n-g-PSSs with E and the corresponding partition function (Z_{nE}^{g}) are the same as Eqs.E1-2.

By expressing s_{nk}^{g}, η_n^{g}, and θ at $t = 0$ as $s_{nk}^{g}(0)$, $\eta_n^{g}(0)$, and $\theta(0)$, respectively, we obtain,

$$s_{nk}^{g}(0)=\frac{1}{Z_n^g}\sum_{\sigma_1\cdots\sigma_n}\sigma_k\exp\left[\varpi\sum_{i=1}^{n-1}\sigma_i\sigma_{i+1}+\theta^E\sum_{j=1}^{n}\sigma_j\right]+O(E^2) \tag{I2}$$

Let the deviation of $s_{nk}^g(0)$ from s_{nk}^{ge} induced by E_0 be $\delta_{nk}^g(0)$, i.e. $\delta_{nk}^g(0)\equiv s_{nk}^g(0)-s_{nk}^{ge}$, then,

$$\delta_{nk}^g(0)=\frac{\partial s_{nk}^g(0)}{\partial E_0}E_0=\frac{\partial s_{nk}^g(0)}{\partial\theta^E(0)}\left[\frac{\partial\theta(0)}{\partial E_0}+\frac{\mu}{k_BT}\right]E_0$$

$$=\frac{\partial s_{nk}^g(0)}{\partial\theta^E(0)}\left[\frac{\mu}{\varepsilon_0}\frac{\partial\eta_n^g(0)}{\partial E_0}\frac{A_n^g}{T}+\frac{C_w}{N_0T}\right]\frac{\varepsilon_0E_0}{\mu}$$

and from $\left.\frac{\partial s_{nk}^g(0)}{\partial\theta^E(0)}\right|_{E_0\to0}=\frac{\partial s_{nk}^{ge}}{\partial\theta_e}=\frac{\partial s_{nk}^{ge}}{\partial\gamma_e}\frac{\partial\gamma_e}{\partial\theta_e}=\left(1-{\gamma_e}^2\right)\frac{\partial s_{nk}^{ge}}{\partial\gamma_e}$, we get,

$$\delta_{nk}^g(0)=\left(1-{\gamma_e}^2\right)\left(\frac{A_n^g}{T}\frac{\chi_s^{ng}}{n}+\frac{C_w}{N_0T}\right)\frac{\partial s_{nk}^{ge}}{\partial\gamma_e}\frac{\varepsilon_0E_0}{\mu} \tag{I3}$$

By substituting this equation into Eq.H23, we obtain the following equations that V_{nn}^{gk} $(k=1,\cdots n)$ obey,

$$\sum_{k=1}^{n}\begin{bmatrix}v_{n1}^{gk}\\ \vdots\\ 1\end{bmatrix}\frac{\mu V_{nn}^{gk}}{\varepsilon_0E_0}=\left(1-{\gamma_e}^2\right)\left(\frac{A_n^g}{T}\frac{\chi_s^{ng}}{n}+\frac{C_w}{N_0T}\right)\begin{bmatrix}\partial s_{n1}^{ge}/\partial\gamma_e\\ \vdots\\ \partial s_{nn}^{ge}/\partial\gamma_e\end{bmatrix} \tag{I4}$$

Therefore, for $t\geq0$, the polarization (P_n^g) of n-g-PSSs with t is,

$$P_n^g=\mu\sum_{i=1}^{n}\delta_{ni}^g=\varepsilon_0E_0\sum_{k=1}^{n}\Delta_{nk}^g e^{-t/\tau_{nk}^g} \tag{I5}$$

and,

$$\Delta_{nk}^g\equiv\frac{V_{nn}^{gk}}{\varepsilon_0E_0}\sum_{i=1}^{n}v_{ni}^{gk} \tag{I6}$$

From Eqs.I5-6, the linear complex permittivity (χ_n^{g*}) of n-g-PSSs is,

$$\chi_n^{g*}=\chi_n^{g\prime}-i_c\chi_n^{g\prime\prime}=\sum_{k=1}^{n}\frac{\Delta_{nk}^g}{1+i_c\omega\tau_{nk}^g} \tag{I7}$$

Obviously,

$$\chi_s^{ng}=\sum_{k=1}^{n}\Delta_{nk}^g \tag{I8}$$

Fig.I1 shows that, compared with Δ_{nn}^g, Δ_{nk}^g $(k=1,\cdots n-1)$ are much smaller, and by Eq.I8, we get,

$$\chi_n^{g*}=\chi_n^{g\prime}-i_c\chi_n^{g\prime\prime}\approx\frac{\chi_s^{ng}}{1+i_c\omega\tau_{nn}^g} \tag{I9}$$

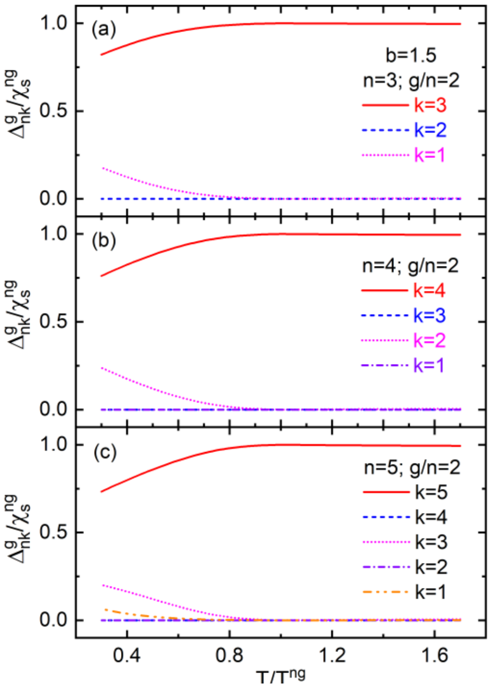

Fig.I1 $\Delta_{nk}^g/\chi_s^{ng}$ $(k=1,\cdots n-1)$ of n-g-PSSs with $b=1.5$ vs T for $g/n=2$ and $n=3,4,5$.

Appendix J: Thermal-strain of RFEs

At present, the Burns transformation of the high-temperature thermal-strain (s_{kl}^T, $k,l=1,2,3$) in RFEs is explained based on the macroscopic quadratic-electro-strictive effect[14,42], i.e. the crystal strain (s_{kl}^{QE}) induced by the spontaneous-polarization components (P_s^i, $i=1,2,3$) is,

$$s_{kl}^{QE}=\sum_{i,j=1}^{3}q_{kl}^{ij}P_s^iP_s^j \tag{J1}$$

where q_{kl}^{ij} is the quadratic electro-strictive coefficient.

So, the thermal-strains (s_{kl}^T) of RFEs is,

$$s_{kl}^T=s_{kl}^0+s_{kl}^{QE} \tag{J2}$$

and s_{kl}^0 is the thermal-strain caused by the non-harmonic portion of the interaction that constructs crystal lattices[14,42].

When the temperature is high enough, s_{kl}^0 satisfies the linear relationship with T, and we get,

$$s_{kl}^T-s_{kl}^0(T_r)=\alpha_{kl}(T-T_r)+s_{kl}^{QE} \tag{J3}$$

Among them, T_r is a reference temperature, α_{kl} is the high-temperature coefficient of thermal-

expansion caused by the non-harmonic interaction, and it is nearly independent of T. Based on Eqs.J1-3, Cross[1] thought that the deviation of s_{kl}^{T} from the high-temperature linear behavior in RFEs is due to the emergence of PNRs[14-18,44-47] first proposed by Burns et al.[14,42].

It is conceivable that the variation in the local-interaction (LI) energy of the nearest-neighbor PS pairs inevitably induce a change of the relative positions of the ions in the unit cell, i.e. the local-distortion (LD) of crystal lattices, which is called as the LI-LD coupling here. It can also be expected that the LI-LD coupling is weak at high-temperature, and under the linear LI-LD coupling approximation, the local distortion (s_{kl}^{ij}) caused by the LI between i[th]- and nearest-neighbor j[th]-PS is,

$$s_{kl}^{ij}=c_{kl}\sigma_i\sigma_j r_i^{(\phi)}r_j^{(\phi)} \tag{J4}$$

where c_{kl} is the coefficient of LI-LD coupling.

Then, the strain (s_{kl}^{LI-LD}) of RFEs related to the LI-LD coupling is,

$$s_{kl}^{LC-LD}\approx\frac{1}{N}\sum_{ij}^{\{nn\}}s_{kl}^{ij}=-c_{kl}\frac{u_{ps}}{J} \tag{J5}$$

and the high-temperature s_{kl}^{T} of RFEs is,

$$s_{kl}^{T}-s_{kl}^{0}(T_r)\approx\alpha_{kl}(T-T_r)-c_{kl}\frac{u_{ps}}{J} \tag{J6}$$

It is worth pointing out that, based on the Weiss, i.e. the single-PS, mean-field of 3D-Ising model[58,59], $u_{ps}\approx-3J\left(\frac{P_s}{N_0\mu}\right)^2$, and it can be obtained from Eq.J6,

$$s_{kl}^{T}-s_{kl}^{0}(T_r)\approx\alpha_{kl}(T-T_r)+3c_{kl}\left(\frac{P_s}{N_0\mu}\right)^2 \tag{J7}$$

which is in agreement with Eq.J3. This suggest that the explanation of the Burns transformation of s_{kl}^{T} in RFEs based on the macroscopic quadratic-electrostrictive effect only considers η_{ne}^{g} [14,42] but does not ζ_{ni}^{ge}, as compared with (Eq.J6)

Appendix K: Refractive-index of RFEs

Currently, the Burns transformation of the high-temperature refractive-index (n_{kl}, $k,l=1,2,3$) in RFEs is explained by the macroscopic Kerr (quadratic-electro-optic) effect[14,42], i.e. the refractive-index (n_{kl}^{KE}, $k,l=1,2,3$) induced by P_s^i ($i=1,2,3$) is,

$$n_{kl}^{KE}=\sum_{i,j=1}^{3}K_{kl}^{ij}P_s^iP_s^j \tag{K1}$$

where K_{kl}^{ij} is the Kerr coefficient.

So, n_{kl} of RFEs is,

$$n_{kl}=n_{kl}^{0}+n_{kl}^{KE} \tag{K2}$$

and n_{kl}^{0} is the refraction index unrelated to the interaction between PSs[14,42].

At high-temperature, n_{kl}^{0} meets the linear relationship with T, and,

$$n_{kl}-n_{kl}^{0}(T_r)=b_{kl}(T-T_r)+n_{kl}^{KE} \tag{K3}$$

Among them, b_{kl} is the high-temperature thermo-optic coefficient. Based on Eqs.K1-3, Burns et al.[14,42] considered for the first time that the deviation of n_{kl} of RFEs to the high-temperature linear behavior is caused by the emergence of PNRs on cooling[44-47].

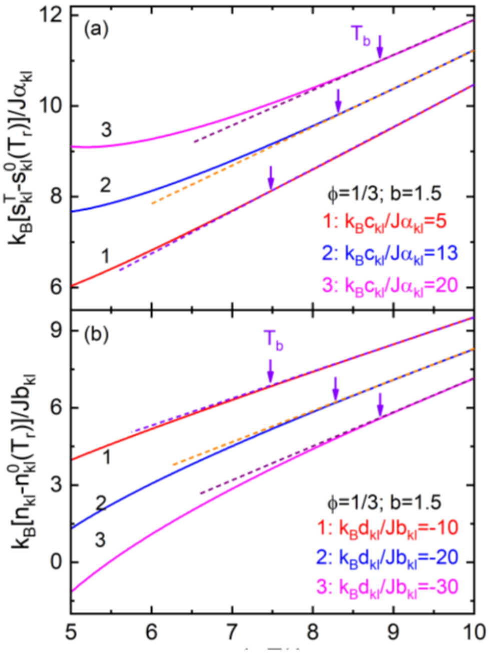

Fig.K1 s_{kl}^{T} (a) and n_{kl} (b) of 3D-ERSIM with $\phi=1/3$ and $b=1.5$ vs T for serial c_{kl}/α_{kl} and d_{kl}/b_{kl}.

The LD of crystal lattices related to the LI will also induce the change of the local-electron-clouds (LE) in the lattices, which is abbreviated as LI-LE coupling. The LI-LE coupling will also lead to the change in the local permittivity of RFEs at optical frequency, and thus in the local refractive-index. It can also be expected that the LI-LE coupling is weak at high-temperature, and under the linear LI-LE coupling approximation, the resulting local refractive-index (n_{kl}^{ij}) by the LI between the ith- and the nearest-neighbor j[th]-PS is,

$$n_{kl}^{ij}=d_{kl}\sigma_i\sigma_j r_i^{(\phi)}r_j^{(\phi)} \tag{K4}$$

where d_{kl} is the LI-LE coupling coefficient.

The refractive-index (n_{kl}^{LI-LC}) of RFEs due to LI-LE coupling is,

$$n_{kl}^{LI-LC} \approx \frac{1}{N}\sum_{i,j}^{\{nn\}} n_{kl}^{ij} = -d_{kl}\frac{u_{ps}}{J} \tag{K5}$$

and the high-temperature n_{kl} is,

$$n_{kl} - n_{kl}^{0}(T_r) \approx b_{kl}(T - T_r) - d_{kl}\frac{u_{ps}}{J} \tag{K6}$$

Also according to the internal energy results of the Weiss mean-field of 3D-Ising model (App. J) and Eq.K6, we obtain,

$$n_{kl} - n_{kl}^{0}(T_r) \approx b_{kl}(T - T_r) + 3d_{kl}\left(\frac{P_s}{N_0\mu}\right)^2 \tag{K7}$$

which agrees with Eq.K3. This indicate that the interpretation of the Burns transition of n_{kl} in RFEs based on the macroscopic Kerr effect only considers η_{ne}^{g} but does not ζ_{ni}^{ge}, as compared with (Eq.K6), too.
